# XPOT Disruption Suppresses TNBC Growth through Inhibition of Specific tRNA Nuclear Exportation and TTC19 Expression to Induce Cytokinesis Failure

**DOI:** 10.7150/ijbs.85006

**Published:** 2023-10-24

**Authors:** Huijuan Dai, Xiaomei Yang, Xiaonan Sheng, Yaohui Wang, Shan Zhang, Xueli Zhang, Lipeng Hu, Zhigang Zhang, Xinrui Dong, Wenjin Yin, Linli Yao, Jinsong Lu

**Affiliations:** 1 Department of Breast Surgery, Renji Hospital, School of Medicine, Shanghai Jiaotong University, Shanghai, 200127, China.; 2 State Key Laboratory of Systems Medicine for Cancer, Shanghai Cancer Institute, Renji Hospital, Shanghai Jiao Tong University School of Medicine, Shanghai, 200127, China.

**Keywords:** triple negative breast cancer, XPOT, tRNA nucleo-cytoplasmic transport, mRNA translation, cytokinesis

## Abstract

Transfer RNAs (tRNAs) impact the development and progression of various cancers, but how individual tRNAs are modulated during triple-negative breast cancer (TNBC) progression remains poorly understood. Here, we found that XPOT (Exportin-T), a nuclear export protein receptor of tRNAs, is associated with poor prognosis in breast cancer and directly orchestrates the nuclear export of a subset of tRNAs, subsequently promoting protein synthesis and proliferation of human TNBC cells. XPOT knockdown inhibited TNBC cell proliferation *in vitro*, and RNA-seq indicated that XPOT is involved in the completion of cytokinesis in TNBC cells. High-throughput sequencing of tRNA revealed that XPOT specifically influenced a subset of tRNA isodecoders involved in nucleocytoplasmic trafficking, including tRNA-Ala-AGC-10-1. Through codon preferential analysis and protein mass spectrometry, we found that XPOT preferentially transported nuclear tRNA-Ala-AGC-10-1 to the cytoplasm, driving the translation of TPR Repeat Protein 19 (TTC19). TTC19 is also indispensable for cytokinesis and proliferation of TNBC cells. Altogether, these findings provide a novel regulatory translation mechanism for preferential tRNA isodecoder nucleocytoplasmic transport through XPOT, which coordinates the spatial location of specific tRNA and the translation of mRNA to facilitate TNBC proliferation and progression. Targeting XPOT may be a novel therapeutic strategy for treating TNBC.

## Introduction

Triple-negative breast cancer (TNBC), with no estrogen receptor (ER), progesterone receptor (PR), or human epidermal growth factor receptor 2 (HER2) expression, has the highest rate of metastasis, relapse, and death among all breast cancer subtypes, accounting for 15-20% of all patients with breast cancer 1. Although several studies have focused on TNBC, the biological mechanisms underlying its development and progression remain unclear. Therefore, there is a high demand for identifying potential molecular mechanisms and effective biomarkers as well as designing individualized targeted therapies for TNBC.

Transfer RNAs (tRNAs), 73-92 nucleotides in length, are a class of small RNAs that play key roles in protein translation by carrying cognate aminoacyl amino acids to ribosomes 2, 3. Their regulation involves multiple steps including synthesis, nuclear modification, aminoacylation, and spatial transportation 4. Different tRNA dysfunctions play oncogenic roles in various cancers, including breast cancer. Tavazoie et al. reported that the overexpression of a specific tRNA-Glu-UUC can boost the invasive and metastatic capacity of breast cancer by increasing its cognate codon usage for unique gene translation 5. Subsequent studies have shown that increasing tRNA^Met^ and tRNA^Leu^ abundance can also promote the growth of breast cancer cells 6, 7. Increasing tRNA transcripts driven by telomerase reverse transcriptase (TERT) in TNBC samples can lead to the upregulated biosynthesis of proliferation-related proteins 8. In addition, tRNA^His^-derived fragments can promote breast cancer cell proliferation 9 and aberrant methylation of cytoplasmic tRNA^His^ in MCF7 cells leads to dysfunction 10. However, the potential roles and underlying mechanisms of tRNA spatial trafficking from the nucleus to cytoplasm in breast cancer, especially in TNBC, have not yet been explored.

The transport of macromolecules, including proteins and nucleic acids, between the nucleus and cytoplasm is a key step in cell signal transduction and is critical for regulating diverse cell biological functions 11. The transportation process is mainly mediated by the karyopherin family, which is composed of importin and exportin proteins. Dysregulated expression or dyslocalization of karyopherin has been observed in numerous cancers and contributes to sustained cancer cell proliferation, resistance to apoptosis, and immune escape 11, 12. Cancer cells are more susceptible to nuclear transporter inhibitors than normal cells, owing to their high metabolism and rapid proliferation rates 13, 14. The karyopherin superfamilies are considered as extremely promising targets for cancer therapy 15. XPOT is an important karyopherin member that specializes in the nucleocytoplasmic trafficking of tRNAs to participate in protein translation for cell growth and metabolism 16, 17. XPOT mediates the subcellular localization of tRNA and responds to nutritional stress by activating the mTORC1 pathway and inhibiting autophagy in human fibroblasts 18. Deficiency of the XPOT homologue, los1, causes decreased nuclear export of tRNA and subsequent accumulation of tRNA in the nucleus, resulting in DNA damage and abnormal cell cycle regulation in yeast cells 19. While several studies have revealed XPOT overexpression in mesothelioma, leukemia HL-60 cells, and hepatocellular carcinoma 20-23, the exact molecular mechanisms of XPOT in these tumors remain elusive, especially whether and how XPOT modulates tumor progression through tRNA nuclear export. Thus, it is of great significance to explore the function and regulatory mechanisms of the tRNA mediator XPOT in TNBC.

In this study, we found that XPOT was associated with poor prognosis in TNBC. *In vivo* and *in vitro* experiments have shown that the inhibition of XPOT could decrease TNBC growth. According to tRNA high-throughput sequencing analysis, XPOT knockdown preferentially decreased the nuclear export of tRNA-Ala-AGC-10-1. Further experimental results from protein mass spectrometry, codon priority analysis, and transcriptome sequencing indicated that XPOT knockdown reduced the translation of the cytokinesis gene TTC19. Collectively, our results revealed that XPOT could potentially regulate the cytokinesis and proliferation of TNBC cells by selectively transporting specific nuclear tRNAs to the cytoplasm. Therefore, XPOT, as a key regulator of tRNA transportation, may be a promising novel prognostic biomarker and potential therapeutic target for patients with TNBC.

## Results

### High XPOT expression predicts shorter overall survival for patients with TNBC

**Figure 1:**
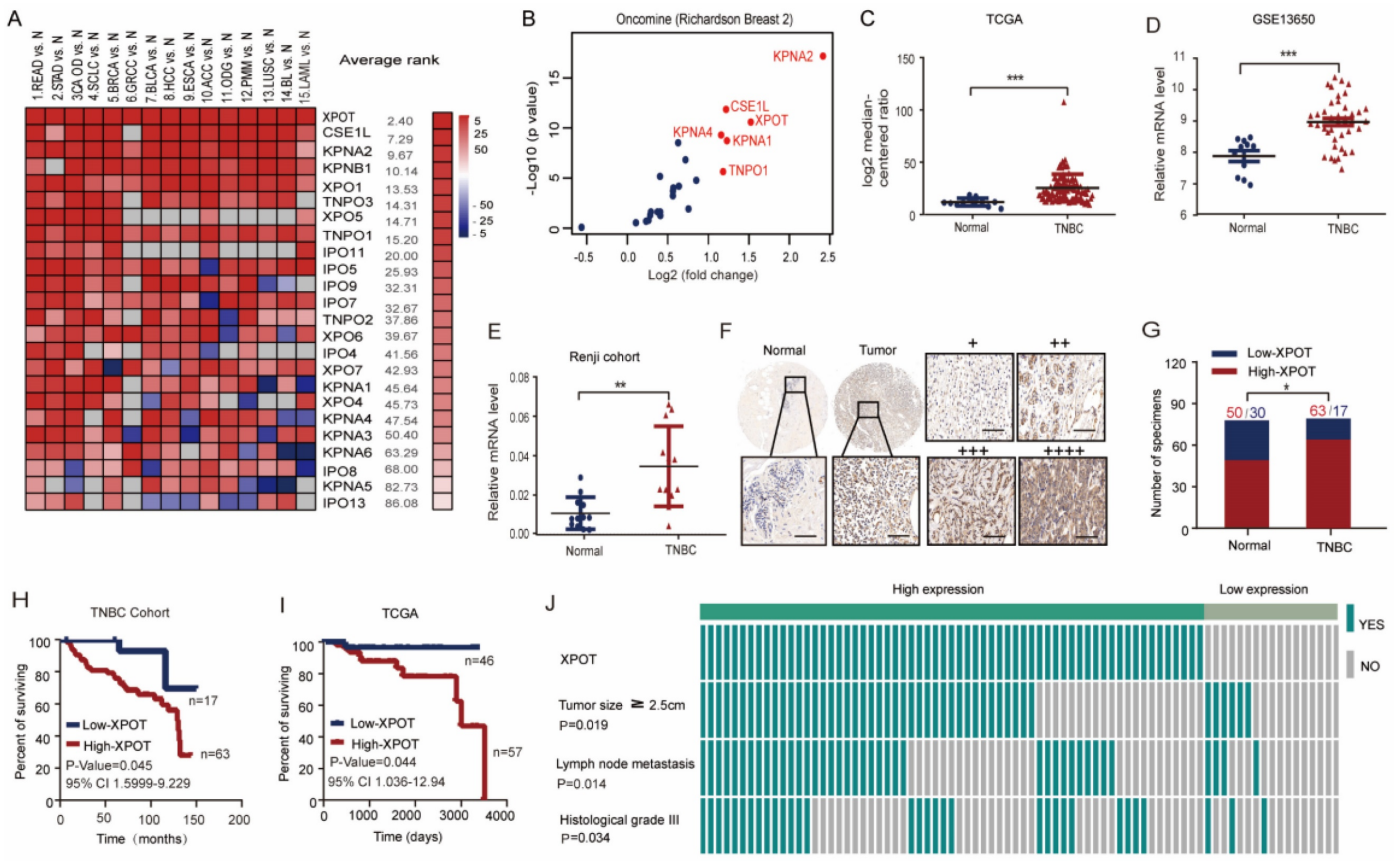
** High XPOT expression predicts poor survival rates in patients with TNBC.** (A) Expression of all 25 karyopherins in 15 cancer datasets from Oncomine. Full information about these cancers is stated in the supplementary material. Karyopherin genes are listed in descending order using the mean rank across each of the analyses. Square lattice color intensity was defined by the gene rank percentile for a specific analysis. (B) Differential expression of 25 karyopherins in a breast cancer dataset from Oncomine is depicted using a scatter plot. (C-E) Analysis of XPOT mRNA level in TNBC tumor tissues compared with normal tissues in TCGA dataset (C), GEO dataset (D), and Renji cohort (E). The levels were compared using Student's t test. (F) Representative images of XPOT immunohistochemistry staining in TNBC and its normal tissues. Scale bar is 50 μm (left panel); Representative images of XPOT staining with different intensity. “+” and “++” are designated as low XPOT expression; “+++” and “++++” represent high expression. Scale bar is 50 μm (right panel). (G) Statistical comparison of XPOT expression level between TNBC and normal tissues based on the immunohistochemical staining (Chi-square test). (H) Kaplan-Meier plot of overall survival for patients with TNBC with low or high XPOT expression based on tissue microarray detection (P=0.045, log rank test). (I) Kaplan-Meier plot of overall survival for patients with TNBC in TCGA dataset (P=0.044, log rank test). (J) Correlation analysis of XPOT expression with tumor size, lymph node metastasis, and histological grade of patients in TNBC tissue microarray (Fisher's exact test).

We initially evaluated all 25 karyopherins across 15 analyses consisting of 16 cancer microarray datasets using Oncomine data-mining. XPOT was found to be the most obviously upregulated karyopherin transport receptor among the 15 cancers (Fig. 1A). The association of the six top-ranked karyopherins (KPNA2, XPOT, CSEL1, KPNA1, KPNA4, and TNPO1) in the breast cancer datasets from Oncomine (Fig. 1B) was analyzed using a Kaplan-Meier Plotter. High XPOT expression predicted worse overall survival in breast cancer, especially in TNBC (Fig. S1B). Through analysis of The Cancer Genome Atlas (TCGA) dataset, the Gene Expression Omnibus (GEO) dataset (GSE13650), and our Renji Hospital cohort, we found that the expression of XPOT mRNA in TNBC tissues was much higher than that in normal tissues (Fig. 1C-E). These results were further validated by immunohistochemical analysis of 80 pairs of samples in the TNBC tissue microarrays (TMA) (Fig. 1F-G). Importantly, high XPOT levels were closely associated with poor survival in TNBC in the public TCGA dataset (P=0.044) and in TNBC TMA (p=0.045) (Fig. 1H and 1I). Multivariate Cox regression analysis showed that XPOT was an independent prognostic factor for overall survival in TNBC based on TMA and TCGA data (Fig. S2A). High XPOT expression was also associated with more aggressive biological characteristics such as larger tumor size (P=0.019), more lymph node metastasis (P=0.014), and higher histological grade (P=0.034) (Fig. 1J).

### XPOT knockdown suppresses TNBC growth through impairing cytokinesis and increasing apoptosis

**Figure 2:**
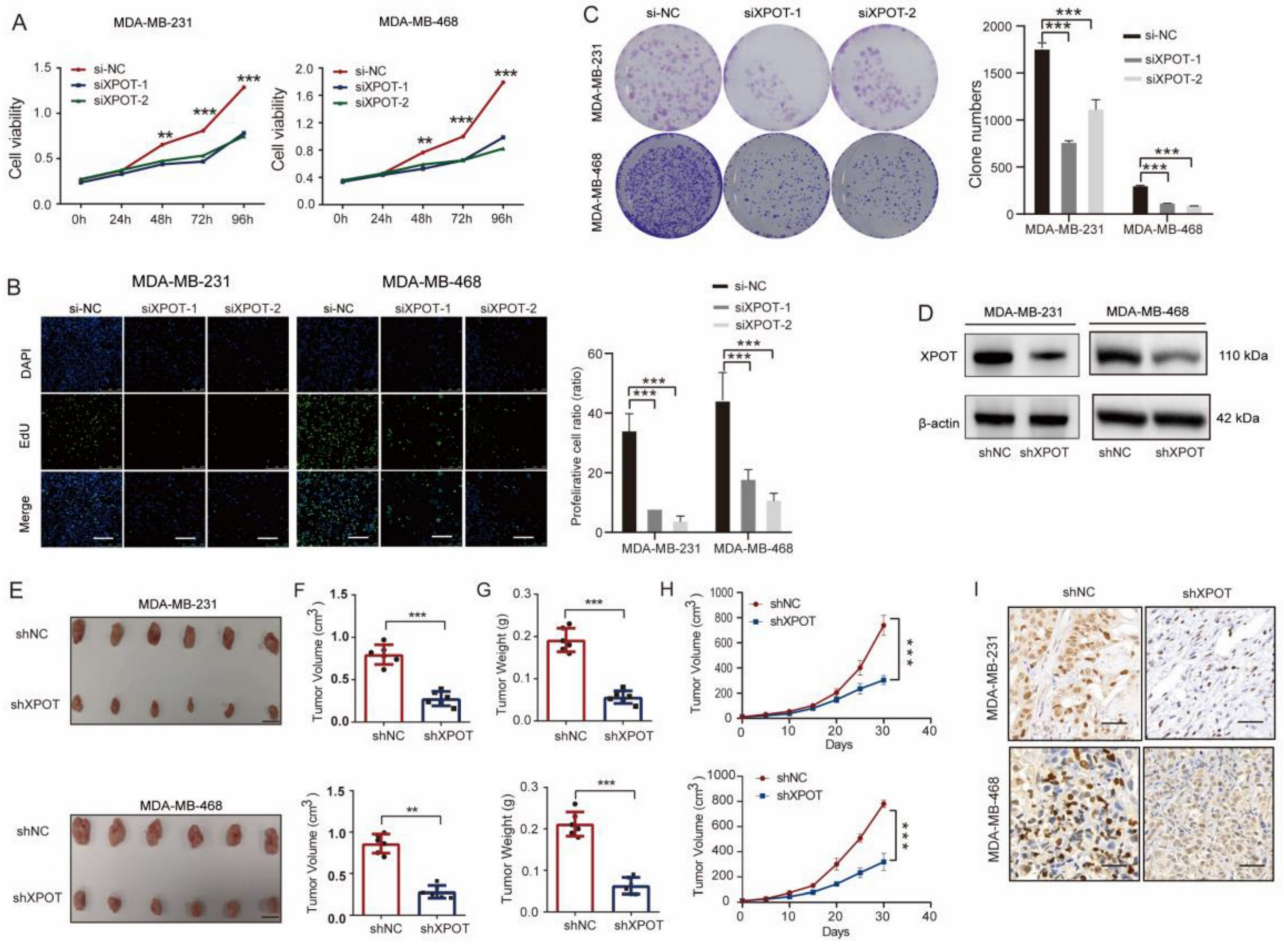
** XPOT facilitates TNBC cell growth *in vitro* and *in vivo*.** (A) CCK8 assay of MDA-MB-231 and MDA-MB-468 cells after XPOT knockdown by siRNA. Results are exhibited as means ± SD of the OD_450_ value (Student's t test). (B) EdU assay of MDA-MB-231 or MDA-MB-468 cells after XPOT depletion by siRNA. Scale bar indicates 50 μm. Values are means ± SD (Student's t test). (C) Representative images of colony formation assay using MDA-MB-231 and MDA-MB-468 cells after XPOT interference. Values are means ± SD (Student's t test). (D) Stable knockdown of XPOT using short hairpin (sh) RNA in MDA-MB-231 and MDA-MB-468 cells by western blotting. (E) The right back flank of each naked nude mouse was treated with 2×10^6^ shNC or shXPOT/MDA-MB-231 and MDA-MB-468 cells, and the tumors were dissected at 30 days for measurement (n=6). Scale bar represents 10 mm. (F, G, and H) Tumor volumes, tumor weights, and survival curves of xenograft tumors from the shNC and shXPOT groups in both MDA-MB-231 and MDA-MB-468 cells. Values are presented as means ± SD (Student's t test). (I) Representative immunohistochemical images of Ki67 staining in subcutaneous xenograft tumor tissues from mice injected with shNC, shXPOT/MDA-MB-231, or MDA-MB-468 cells. Scale bar indicates 50 μm. **P < 0.01; ***P< 0.001

Initially, we detected the expression of XPOT in several TNBC cell lines and in a normal control breast epithelial cell line, HBL100, by western blotting. XPOT protein expression in TNBC cells was higher than that in control cells (Fig. S3A). Based on their differential expression, two TNBC cell lines, MDA-MB-231 and MDA-MB-468, were selected for further experiments. In these two cell lines, XPOT was localized to both the cytoplasm and nucleus (Fig. S4). For further functional experiments, XPOT depletion in MDA-MB-231 and MDA-MB-468 cells by small interfering RNA (siRNA) was validated using quantitative RT-PCR analysis (Fig. S3B). We observed that the proliferation of MDA-MB-231 and MDA-MB-468 cells was inhibited after XPOT downregulation using the CCK8, EdU, and colony formation assays (Fig. 2A-C). To further verify the effect of XPOT on TNBC growth *in vivo*, we constructed a lentivirus-based stable RNA interference vector using the XPOT-siRNA2 interference sequence. The knockdown efficiency of Lenti-shXPOT-transfected MDA-MB-231 and MDA-MB-468 cells was demonstrated by western blotting (Fig. 2D), which revealed that XPOT knockdown prominently inhibited tumor growth in the xenograft tumor model established with MDA-MB-468 cells (Fig. 2E-G). Consistently, IHC staining of Ki67 showed that tumor cell proliferation was decreased in shXPOT xenografts compared to that in controls (Fig. 2H).

**Figure 3:**
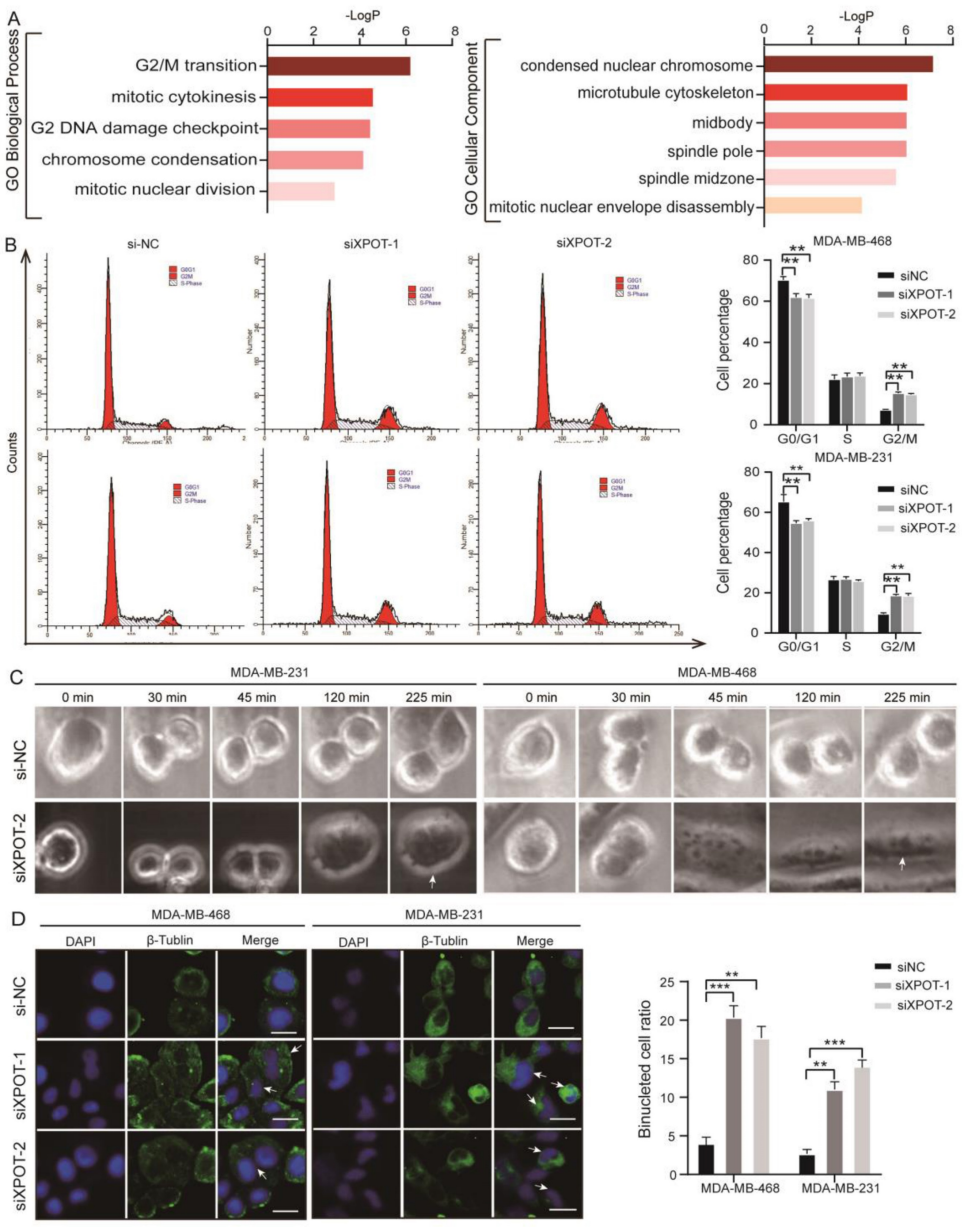
** XPOT ensures successful cytokinesis and resists apoptosis of TNBC cells.** (A) Gene ontology analysis based on DEGs in RNA-seq of si-NC and si-XPOT MDA-MB-468 cells in which cytokinesis-related pathways were significantly enriched was conducted. (B) Cell cycle analysis for si-NC and si-XPOT group in MDA-MB-468 and MDA-MB-231 cells. Representative images and quantitative cell count in each phase are shown. Values are means ± SD (Student's t test). (C) Selected frames from Live Cell Imaging of si-NC and si-XPOT/MDA-MB-468 cells. Binucleated cell is indicated by white arrowhead. (D) Representative immunofluorescence staining of β-tubulin and DAPI in si-NC and si-XPOT/MDA-MB-468 cells or MDA-MB-231 cells (left panel). Binucleated cell is indicated by white arrowhead. The binucleated cells ratio was analyzed, as shown on the right panel. Scale bar represents 20 μm. Values are mean ± SD (Student's t test). SD, standard deviation; n, number; GAPDH, glyceraldehyde-3-phosphate dehydrogenase; DAPI, 40,6-diamidino-2-phenylindole; FITC, fluorescein isothiocyanate; PI, propidium iodide; si, small interfering RNA. *P< 0.05; **P < 0.01; ***P< 0.001.

To further explore the underlying molecular mechanism of XPOT in TNBC cells, we performed RNA sequencing of MDA-MB-468 cells transfected with si-NC and si-XPOT. Gene Ontology (GO) analysis of differentially expressed genes (DEGs) showed that genes related to cell mitotic processes, especially those linked to chromosomes and the spindle apparatus, were enriched (Fig. 3A). This suggested that XPOT is involved in mitosis. To determine which phase of mitosis is affected by XPOT, we performed cell cycle analysis and found that the percentage of cells in the G2/M phase increased significantly after XPOT knockdown in MDA-MB-231 and MDA-MB-468 cells, whereas the percentage of cells in the G0/G1 phase decreased, indicating that the cell cycle was arrested at the G2/M stage after XPOT knockdown (Fig. 3B). Therefore, we speculated that XPOT might influence the final stage of mitosis.

We then performed time-lapse live cell imaging and immunofluorescence analysis to observe the binucleated cells. The results showed that the binucleated cell ratio increased after XPOT knockdown (Fig. 3C and 3D). Cytokinesis mostly occurs in the anaphase and telophase of the cell cycle, with the features of mounting binucleated or multinucleated cells. Thus, the above results imply that XPOT may play an essential role in TNBC cell cytokinesis. In addition, cell apoptosis was detected by flow cytometry, and we found that the proportion of apoptotic cells increased after XPOT depletion compared to that in the control cells (Fig. S5). Generally, these findings show that the downregulation of XPOT potentially decreases TNBC growth by impairing cytokinesis and promoting apoptosis.

### XPOT preferentially affects tRNA-Ala-AGC-10-1 nucelo-cytoplasmic distribution in TNBC cells

**Figure 4:**
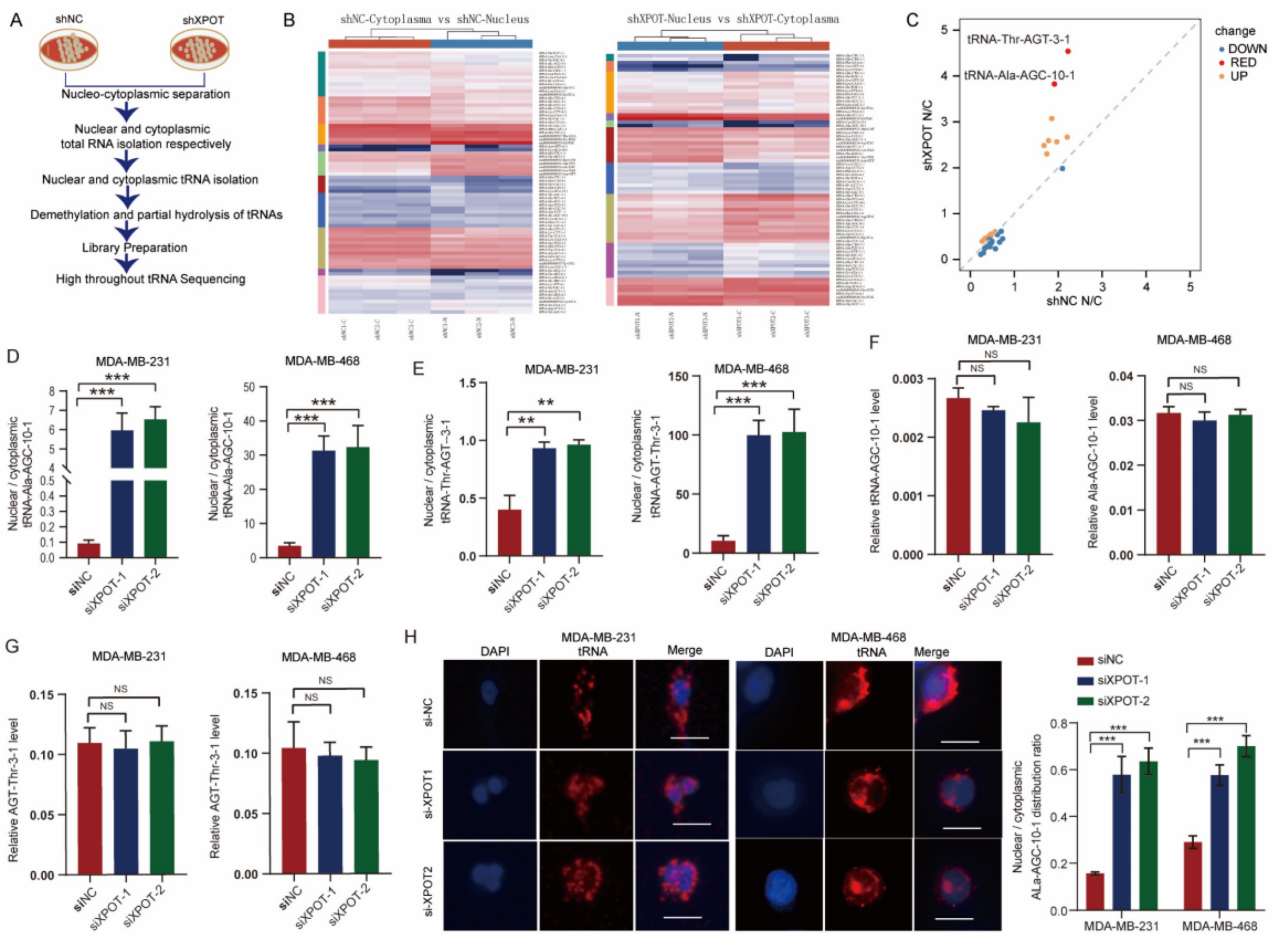
** XPOT enhances specific tRNA-Ala-AGC-10-1 translocation to cytoplasm.** (A) The simple flow diagram of preparation for tRNA sequencing of shNC and shXPOT/MDA-MB-468 cells. (B) Heatmaps of tRNAs distribution in different subcellular compartments (cytoplasm or nucleus) of different groups (shNC or shXPOT) in MDA-MB-468 cells. (C) Nuclear/cytoplasmic ratio of differentially distributed tRNA in shNC and shXPOT MDA-MB-468 cells. Red points indicate the tRNAs with the most transition alteration after XPOT inhibition compared with control cells. (D) Nuclear/cytoplasmic tRNA ratio of tRNA-Ala-AGc-10-1 between si-NC and si-XPOT/MDA-MB-231 or MDA-MB-468 cells using qRT-PCR assay (18S as a cytoplasmic internal control and U6 as a nuclear internal control). Values are mean ± SD (n=3; Student's t test). (E) Nuclear/cytoplasmic tRNA ratio of tRNA-Thr-AGT-3-1 between si-NC and si-XPOT/MDA-MB-231 or MDA-MB-468 cells using qRT-PCR assay (18S as a cytoplasmic internal control and U6 as a nuclear internal control). Values are mean ± SD (n=3; Student's t test). (F-G) Total relative tRNA-Ala-AGC-10-1 (F) and tRNA-Thr-AGT-3-1 (G) abundance in MDA-MB-231 and MDA-MB-468 cells using qRT-PCR assay. Values are mean ± SD (n=3; Student's t test). (H) Nuclear/cytoplasmic tRNA ratio in XPOT-knockdown MDA-MB-231 or MDA-MB-468 cells and their control cells using tRNA scope assay. Scale bar represents 20 μm. Representative images of tRNA distribution are shown, and quantitative analysis is presented as means ± SD (n=3; Student's t test). U6, small nuclear RNA U6; *P < 0.05; **P < 0.01; ***P< 0.001. NS, no significance.

XPOT is a nuclear transport receptor responsible for exporting tRNAs from the nucleus to the cytoplasm. To determine the biological mechanism by which XPOT participates in the cytokinesis of TNBC cells, we extracted cytoplasmic and nuclear tRNA from stable XPOT-knockdown MDA-MB-468 TNBC and control cells (Fig. 4A). High-throughput tRNA sequencing was then performed (Fig. 4B). Based on the tRNA sequencing results, we determined the differential subcellular distribution of tRNAs between XPOT-knockdown and control cells according to the ratio of nuclear tRNA/cytoplasmic tRNA (Fig. 4C; differential tRNAs listed in Table 1). The top two tRNAs with upregulated nucleocytoplasmic distribution, tRNA-Ala-AGC-10-1 and tRNA-Thr-AGT-3-1, were selected for further examination. The qRT-PCR assay showed that the knockdown of XPOT decreased the distribution of tRNA-Ala-AGC-10-1 and tRNA-Thr-AGT-3-1 in the cytoplasm of TNBC MDA-MB-468 and MDA-MB-231 cells (Fig. 4D and 4E), whereas the total content of these two tRNAs in the two cells remained unchanged (Fig. 4F and 4G). Next, we used RNAscope to locate endogenous tRNA-Ala-AGC-10-1 in TNBC cells.

Compared with control cells, the nuclear/cytoplasmic fluorescence ratio (N/C) in XPOT knockdown cells was significantly increased in MDA-MB-231 and MDA-MB-468 cells (Fig. 4H). In addition, we mapped all XPOT tRNAs detected in MDA-MB-468 cells to the tRNA database (GtRNAdb; http://gtrnadb.ucsc.edu/) through tRNA-seq and found 15 tRNA-Ala-AGC isodecoders in the MDA-MB-468 cell line (Appendix. S1), whose sequences are displayed in Fig. S6. To further confirm that XPOT mainly affected the nuclear export of tRNA-Ala-AGC-10-1 rather than the other isodecoders, we detected the distribution of the remaining 14 isodecoders using the primers tRNA-Ala-AGC-3 and tRNA-Ala-AGC-1, since primers for tRNA-Ala-AGC-3 and tRNA-Ala-AGC-1 can cover all the remaining isodecoders, as listed on the Arraystar website (https://www.arraystar.com/rtstar-pre-designed-trna-primer-sets/). The results showed that the nucleocytoplasmic distribution and expression of the remaining 14 isodecoders were not significantly different (Fig. S7A-C). To further verify the tRNA bias of XPOT in tRNA transport during TNBC cell growth, tRNA-Gly-CCC-1-1, a tRNA that remained almost unchanged in the tRNA sequencing results, was analyzed (Table 1). There were no significant differences in the nuclear/cytoplasmic ratio or total level of tRNA-Gly-CCC-1-1 between XPOT-knockdown and control MDA-MB-231 or MDA-MB-468 cells (Fig. S7D and 7E). Therefore, these findings imply that XPOT may preferentially affect the nucleocytoplasmic distribution of tRNA-Ala-AGC-10-1 to play its biological roles.

### Impact of XPOT on TTC19 expression may be tRNA-Ala-AGC-10-1-dependent

**Figure 5:**
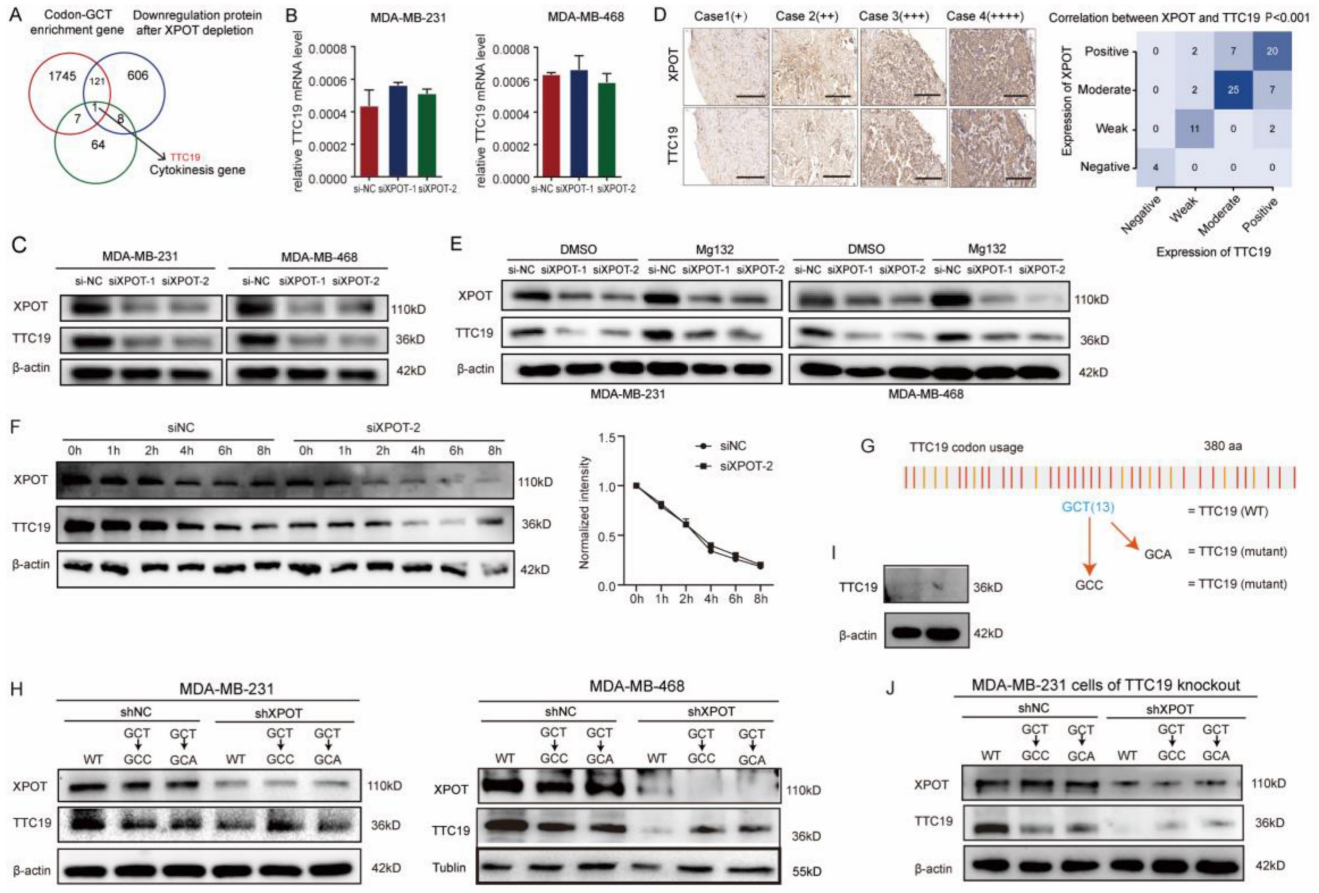
** XPOT regulation of TTC19 expression is codon-dependent.** (A) The Venn diagram of GCT-enriched cytokinesis genes post-transcriptionally regulated by XPOT in TNBC cells. Intersection of 80 cytokinesis genes with codon-GCT-enriched genes and genes translationally regulated by XPOT. Interestingly, we found one cytokinesis-related gene. (B) Relative TTC19 expression at mRNA level after XPOT knockdown mediated by siRNA. Values are mean ± SD (n=3; Student's t test). (C) TTC19 expression at protein level in si-NC and si-XPOT/MDA-MB-231 and MDA-MB-468 cells using western blot assay. The band density was applied to analyze XPOT and TTC19 relative expression to β-actin. Values are means ± SD (n=3; Student's t test). (D) Representative images of immunohistochemistry staining for XPOT and TTC19 by utilizing serial TNBC tissue microarray slice marked with different protein levels. Scale bar indicates 50 μm. The correlation between XPOT and TTC19 expression in the TNBC tissue microarray shown on the below panel is statistically significant (n=80, P<0.001; Fisher exact test). (E) TTC19 expression after proteasome inhibitor MG132 treatment in si-NC and si-XPOT/MDA-MB-231 and MDA-MB-468 cells by western blot analysis. Cells were subjected to siRNA interference for 24 h. We then added MG132 (150 nmol/L) into the cells, and 48 h later, cell lysates were used to perform the western blot assay. The band density was applied to analyze XPOT and TTC19 relative expression to β-actin. Values are means ± SD (N=3; Student's t test). (F) MDA-MB-231 cells expressing si-NC or si-XPOT were treated with CHX (100 mg/mL) for the indicated time points, 0, 1, 2, 4, 6, and 8 h, respectively. MDA-MB-231 cells were transfected with either control siRNA or si-XPOT for 48 h, followed by CHX (100 mg/mL) treatment for the indicated times. The cell extracts were detected by western blot to detect TTC19 expression. (G) Representation of TTC19 coding sequence. (H) TTC19 expression in shNC and shXPOT/MDA-MB-231 or MDA-MB-468 cells that were transfected with wild-type TTC19 plasmid, GCC, or GCA synonymous mutational plasmid. (I) TTC19 knockout efficiency by western blot assay. The TTC19 knockout cell lines were generated using the CRISPR/Cas9 system. (J) The impact of XPOT on TTC19 level. TTC19 knockout MDA-MB-231 cells were transfected with shNC or shXPOT virus to construct XPOT stable knockdown cells. Then above cells were transfected with wild-type TTC19 plasmid, GCC, or GCA synonymous mutational plasmid. The cell extracts were detected by western blot.

Owing to the degeneracy of codons, the anticodon-AGC can identify the codon GCR (GCT, GCC, and GCA). However, the cognate codon of tRNA-Ala-AGC-10-1 is GCT. To explore genes with high GCT abundance, we performed codon usage preference analysis for all transcribed genes in MDA-MB-468 cells. Consequently, 1874 genes were found to have a high content of GCT (GCT score >0.02; GCT score/GCC score >1, GCT score/GCA score >1, Appendix S2). Then, we performed a protein mass spectrometry assay using XPOT-knockdown and control MDA-MB-468 cells to detect the proteins modulated by XPOT and identified 736 downregulated proteins whose transcriptional levels were not altered (Fig. S8). To identify the functionally related target genes of tRNA-Ala-AGC-10-1, we examined the intersection of 80 cytokinesis-related genes with codon-GCT-enriched genes and genes translationally regulated by XPOT. Interestingly, we identified a cytokinesis-related gene, TTC19 (Fig. 5A). Therefore, we postulated that XPOT might affect TTC19 translation, and further experiments were performed to validate this hypothesis. First, the qRT-PCR assay showed that the TTC19 transcript was not affected after XPOT knockdown compared to that in control cells (Fig. 5B). However, western blotting analysis revealed a significant decrease (22-fold reduction) in TTC19 protein expression after XPOT knockdown (Fig. 5C), and immunochemistry staining of serial sections from the TNBC tissue microarray revealed that TTC19 expression was positively correlated with XPOT expression (Fig. 5D). We used a protein proteasome inhibitor (MG132) to determine whether XPOT played a role in TTC19 protein degradation. The results showed that TTC19 degradation in XPOT knockdown cells was comparable to that in control cells (Fig. 5E).

To further verify whether TTC19 expression was GCT-dependent, we constructed a TTC19 coding sequence (CDS) mutant plasmid in which each cognate codon GCT of tRNA-Ala-AGC-10-1 was replaced by the synonymous codon GCC or GCA (Fig. 5F). MDA-MB-231 and MDA-MB-468 cells were transfected with wild-type TTC19 plasmid or mutational plasmid. TTC19 protein expression decreased after XPOT knockdown in wild-type TTC19 plasmid-transfected cells, whereas TTC19 expression was not affected by XPOT knockdown in mutant plasmid-transfected cells (Fig. 5G). We employed CRISPR-Cas9 to knock out TTC19 expression in shNC and shXPOT MDA-MB-231 cells, and the efficiency of which is shown in Figure 5I. We transfected the wild-type TTC19 plasmid or the GCT sequence mutational plasmid into TTC19 shNC and shXPOT knockout MDA-MB-231 cells, and western blot analysis was performed to detect TTC19 expression. TTC19 protein expression decreased after XPOT knockdown in wild-type TTC19 plasmid-transfected cells, while TTC19 expression was not significantly affected by XPOT knockdown in mutational plasmid-transfected cells. The results are shown in Figure 5J. These data indicate that the cytokinetic gene TTC19 may be translationally regulated by XPOT-mediated tRNA-Ala-AGC-10-1 nuclear export in TNBC cells.

### TTC19 is essential for TNBC cell proliferation and cytokinesis completion

**Figure 6:**
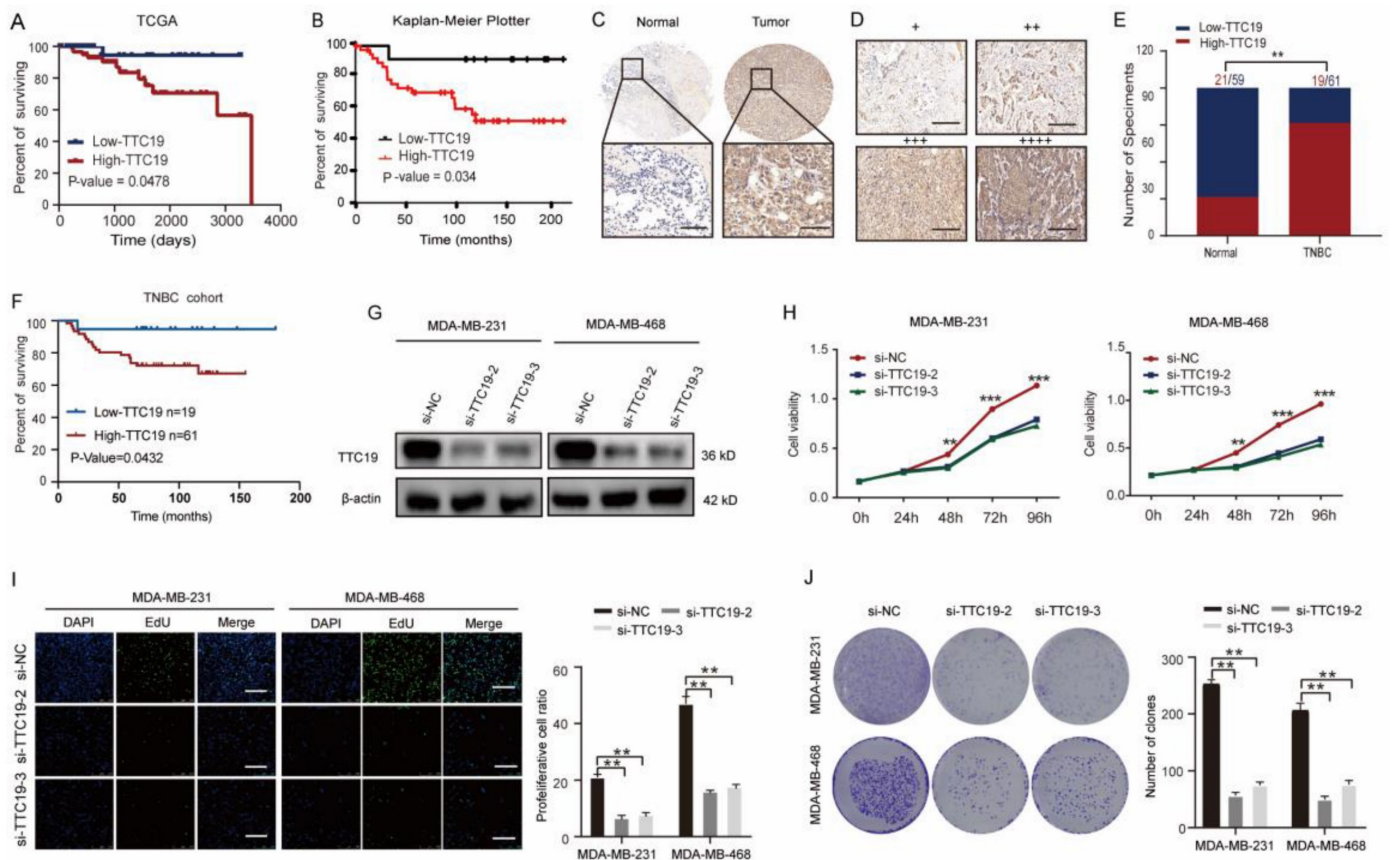
** TTC19 is upregulated in TNBC and promotes proliferation of TNBC cells *in vitro.*** (A) Kaplan-Meier plot of overall survival in patients with TNBC with different TTC19 expression levels from TCGA dataset (P=0.0478, Log rank test). (B) Kaplan-Meier plot for overall survival of patients with TNBC from the online analysis tool Kaplan-Meier Plotter (P=0.034, Log rank test). (C) Representative images of TTC19 immunohistochemistry staining in TNBC and its normal tissues. Scale bar is 50 μm. (D) Representative images of immunohistochemistry about TTC19. + and ++ mean low TTC19 expression level; +++ and ++++ mean high expression level. Scale bar is 50 μm. (E) Statistical comparison of TTC19 expression level between TNBC and normal tissues based on the immunohistochemical staining (Chi-square test). (F) Kaplan-Meier plot of patients with TNBC with low or high TTC19 expression based on TNBC tissue microarray detection (P=0.0275, Log rank test). (G) Western blot analysis of TTC19 expression in TTC19 knockdown and control MDA-MB-231 and MDA-MB-468 cells. (H) CCK8 assay of MDA-MB-231 and MDA-MB-468 cells after TTC19 interfered by siRNA. Results are shown as means ± SD of the OD_450_ value (n=3; Student's t test). (I) EdU assay of MDA-MB-231 and MDA-MB-468 cells after XPOT depletion by siRNA. Scale bar indicates 50 μm. Values are means ± SD (n=3; Student's t test). (J) Representative images and statistical analysis of colony formation assay of MDA-MB-231 and MDA-MB-468 cells after TTC19 knockdown. Values are means ± SD (n=3; Student's t test). **P < 0.01; ***P< 0.001.

TTC19 is involved in cytokinesis, as described on the GeneCards website (https://www.genecards.org/cgi-bin/carddisp.pl?gene=TTC19&keywords=TTC19), but its exact function in cancers, especially in TNBC, remains unknown. To determine the role of TTC19 in TNBC, we investigated the correlation between TTC19 expression and survival in TNBC. High levels of TTC19 mRNA were found to be associated with poor overall survival in TNBC by analyzing TCGA dataset and Kaplan-Meier Plotter (http://kmplot.com/analysis/) (Fig. 6A, B). Through immunohistochemical staining of TNBC TMA, we found that the expression of TTC19 in TNBCs was higher than that in normal tissues (Fig. 6C-E). Higher protein expression of TTC19 was closely associated with larger tumor size, more lymph node metastasis, higher histological grade (Fig. S9), and poor overall survival (Fig. 6F) in patients with TNBC. In addition, we found that the depletion of TTC19 phenocopied the effects of XPOT downregulation on cell proliferation and apoptosis, as detected by CCK8 (Fig. 6H), EdU (Fig. 6I), colony formation assays (Fig. 6J), and Annexin V/PI flowmetry assays (Fig. S10).

**Figure 7:**
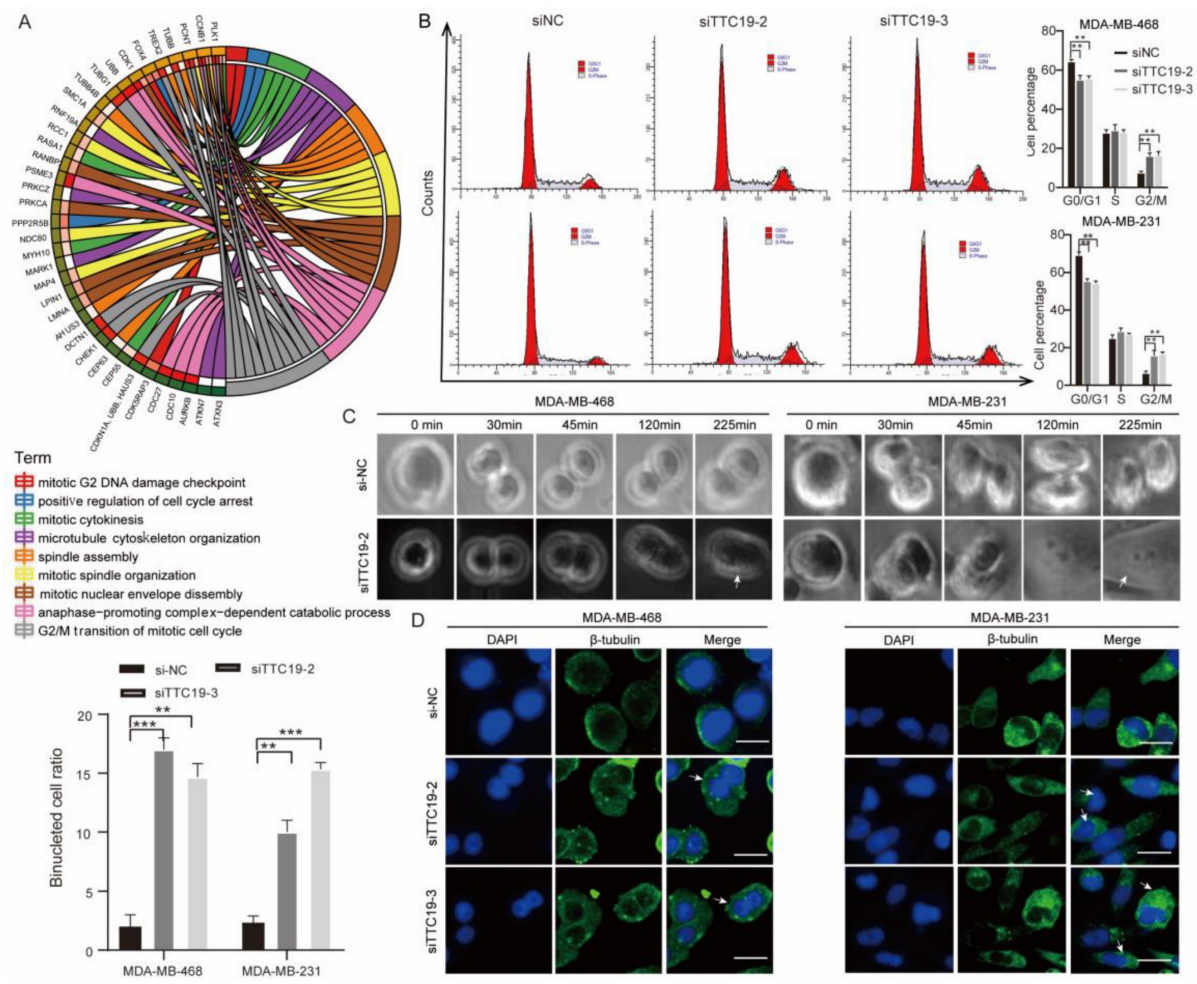
** TTC19 ensures successful TNBC cell cytokinesis.** (A) Circos plot of relationship between DEGs in siTTC19 cells and cytokinesis-related process. (B) Cell cycle analysis of si-NC and si-TTC19 in MDA-MB-468 and MDA-MB-231 cells. Representative images and quantitative cell count in each phase are shown. Values are means ± SD (n=3; Student's t test). (C) Selected frames from Live Cell Imaging of si-NC and si-TTC19/MDA-MB-468 cells. Binucleated cell is indicated by white arrowhead. (D) Representative immunofluorescence staining of β-Tubulin and DAPI displaying binucleated cells. Binucleated cell is indicated by white arrowhead. The binucleated cells ratio was compared between TTC19 knockdown and control in MDA-MB-468 and MDA-MB-231 cells on the left panel. Scale bar represents 20 μm. Values are mean ± SD (n=3; Student's t test). **P < 0.01; ***P< 0.001. DEG, differentially expressed genes.

We then performed mRNA sequencing of the si-NC and siTTC19 MDA-MB-468 cells. Biological process annotation showed that the DEGs were enriched in various cytokinesis-related processes (Fig. 7A). Cell cycle analysis (Fig. 7B), time-lapse live cell imaging (Fig. 7C), and immunofluorescence analysis (Fig. 7D) showed that TTC9 knockdown resulted in cell cycle arrest in the G2/M phase, failure of cytokinesis, and formation of more binuclear cells in both TNBC MDA-MB-231 and MDA-MB-468 cells. These results indicated that TTC19 affects TNBC cell growth by regulating cytokinesis.

### TTC19 expression is indispensable for XPOT-regulated TNBC growth

**Figure 8:**
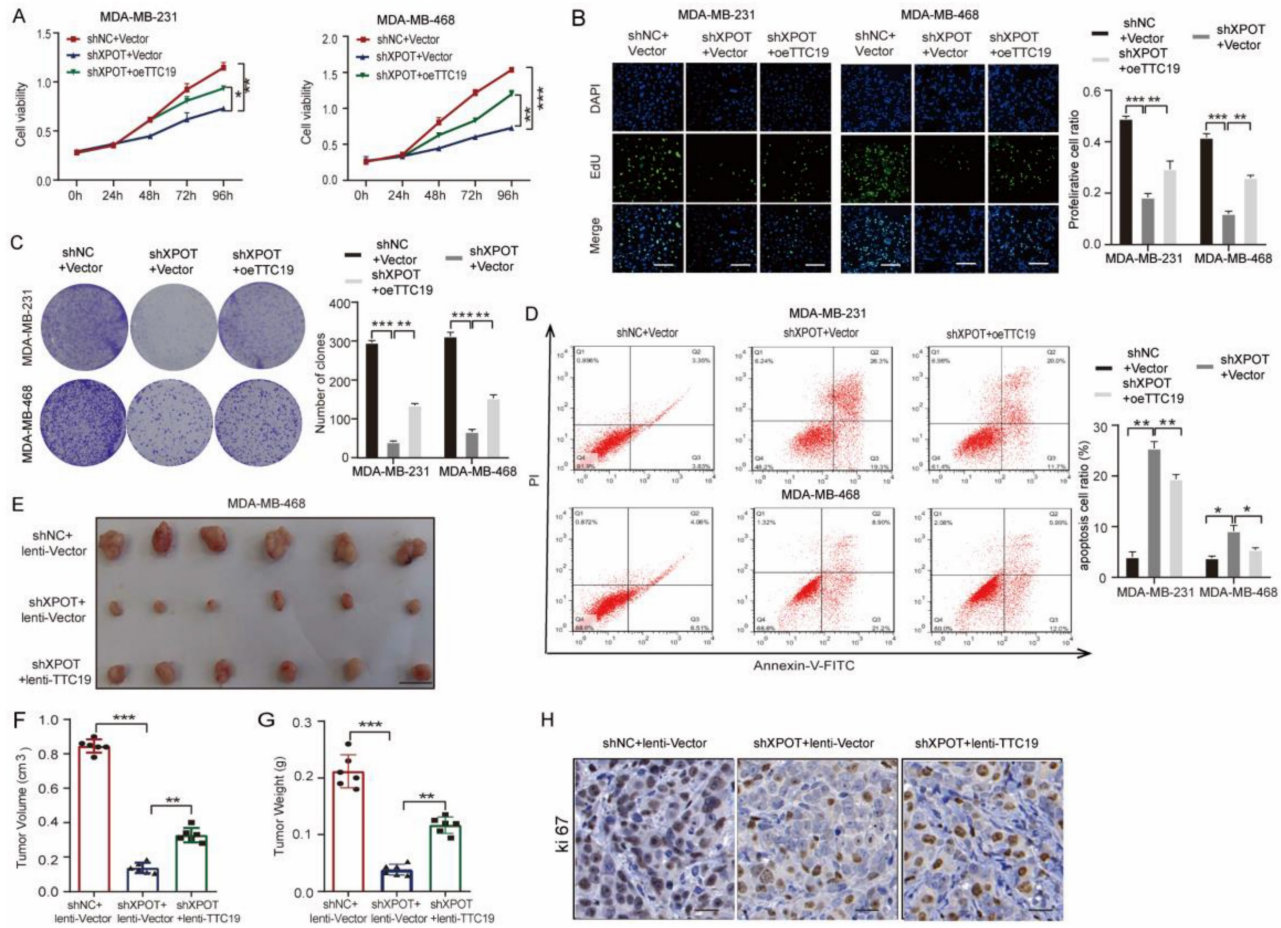
**XPOT promotes TNBC growth partially through driving TTC19 expression.** (A) CCK8 assay of MDA-MB-231 and MDA-MB-468 cells in control and XPOT-knockdown cells transfected with TTC19 overexpression or control vector. Results are shown as means ± SD (n=3; Student's t test). (B) EdU assay of MDA-MB-231 and MDA-MB-468 cells in control and XPOT-knockdown cells transfected with TTC19 overexpression or control vector. Scale bar indicates 50 μm. Values are means ± SD (n=3; Student's t test). (C) Representative images of colony formation assay in control and XPOT-knockdown MDA-MB-231 and MDA-MB-468 cells transfected with TTC19 overexpression or control vector. Values are means ± SD (n=3; Student's t test). (D) Representative images and quantitative apoptotic cells assay in control and XPOT-knockdown MDA-MB-231 and MDA-MB-468 cells transfected with TTC19 overexpression or control vector. Values are mean ± SD (n=3; Student's t test). (E-G) The right back flanks of each naked nude mouse were treated with 2×10^6^ shNC/vector, shXPOT/vector, or shXPOT/oeTTC19/MDA-MB-468 cells. Tumors were dissected for analysis after growing for 30 days (E). Tumor volumes (F) and weights (G) are shown as means ± SD (n = 6 for each group; Student's t test). Scale bar represents 10 mm. (H) Representative immunohistochemistry images of Ki67 staining in subcutaneous xenograft tumor tissue from mice injected with indicated cells. Scale bar indicates 50 μm. *P < 0.05; **P < 0.01; ***P< 0.001.

To explore whether the function of XPOT was dependent on TTC19, we overexpressed TTC19 in shXPOT/MDA-MB-231 and MDA-MB-468 cells by transfecting the plasmid with TTC19 or the vector control. We observed that TTC19 expression recovered moderately after transfection with XPOT-knockdown cells (Fig. S11A). CCK8, Edu, and colony formation assays showed that forced overexpression of TTC19 partially restored the proliferation of MDA-MB-231 and MDA-MB-468 cells, which was inhibited by XPOT depletion (Fig. 8A-C). TTC19 re-expression also partially decreased apoptosis in MDA-MB-231 and MDA-MB-468 cells induced by XPOT knockdown (Fig. 8D). Next, we transfected lentivirus-TTC19 or lentivirus-vector into shXPOT/MDA-MB-468 cells to construct stable cell lines. The western blotting results demonstrated that TTC19 expression recovered moderately (Fig.S11B). Consistently, TTC19 partially restored the growth of TNBC tumors in the subcutaneous xenograft model (Fig. 8E-H). Therefore, we speculated that TTC19 might serve as a downstream protein of XPOT and participate in the regulation of cytokinesis, apoptosis, and proliferation.

## Discussion

XPOT, a member of the importin-β family, moves between the nucleus and cytoplasm and is responsible for tRNA nucleocytoplasmic transportation of tRNA 17, 24. This study is the first to elucidate the oncogenic role of XPOT in promoting TNBC development by ensuring the successful completion of cell cytokinesis. tRNA is a key component of protein translation that carries cognate aminoacyl amino acids to ribosomes 3. We clarified the novel biological function of XPOT through its preferential effect on certain isodecoders of tRNA nucleocytoplasmic distribution according to tRNA high-throughput sequence analysis and found that XPOT knockdown preferentially increased the accumulation of tRNA-Ala-AGC-10-1 in the nucleus, rather than other isodecoders of tRNA-Ala-AGC. We further revealed that XPOT influenced the translation of the cytokinesis-related gene TTC19 by affecting tRNA-Ala-AGC-10-1 nuclear export. We also first revealed that TTC19 might be a tumorigenic gene involved in TNBC growth by guaranteeing the completion of cytokinesis. Furthermore, we demonstrated that TTC19 is essential for the oncogenic role of XPOT in TNBC. Taken together, these data revealed a novel mechanism that XPOT promotes TNBC growth by regulating the tRNA-TTC19 pathway to mediate the completion of TNBC cell cytokinesis (Fig. S12). We delineated a novel role for XPOT via its modulatory effect in protein translation. The knockdown of XPOT or TTC19 inhibited the growth of TNBC cells, indicating that targeting XPOT or TTC19 could be a promising novel therapeutic strategy for TNBC. Previous studies have indicated that XPOT is indispensable for maintaining the physiological functions of normal cells. Herbein et al. reported that XPOT enhanced Nef/eEF1A/tRNA complex transport to the cytoplasm and increased tRNA binding to mitochondrial cytochrome c, causing apoptosis resistance in primary human macrophages 25. Prætorius-Ibba et al. observed that XPOT knockdown led to nuclear accumulation of tRNAs, mTOR activation, and autophagosome formation under nutritional stress conditions in human fibroblasts 18. However, the role and underlying mechanisms of XPOT in breast cancer, especially TNBC, have not been explored. In this study, we found that XPOT overexpression was related to worse survival in TNBC, and inhibition of XPOT suppressed TNBC cell proliferation both *in vitro* and *in vivo*. These results suggested that XPOT may act as an oncogenic gene in TNBC. A previous bioinformatic analysis of public databases partially supported our results, which reported that high XPOT expression could predict worse clinical outcomes 26. However, this study did not analyze the effects of XPOT on TNBC. The intrinsic mechanism by which XPOT enhances the malignant behavior of TNBC requires further investigation. Our study revealed that XPOT knockdown inhibits TNBC cell proliferation by inducing cytokinesis failure. Cytokinesis begins during the anaphase of mitosis, and its failure can lead to cell growth suppression and apoptosis 27, 28. Furthermore, our study revealed a novel underlying mechanism by which XPOT promotes TNBC growth by upregulating downstream cytokinesis gene-TTC19 translation. Additionally, XPOT influenced the expression of tRNA-Ala-AGC-10-1-related genes (P4HA2, ENY2, ELOVL1, etc.) and is regarded as a prognostic factor for poor outcomes in TNBC 29-31. These findings indicate that XPOT plays a crucial role in TNBC progression by modulating different biological processes.

Translation is an important biological process for regulating gene expression in numerous cellular events. Translation-regulated pathways are complex, and tRNAs are indispensable components for codon decoding during protein translation 32, 33. Pavon-Eternod et al. reported that tRNAs are selectively upregulated during breast cancer development, and differential tRNA expression orchestrates crucial oncogenic gene expression for tumor growth. Cancer-related proteins may have high codon usage frequency because their cognate tRNA levels increase in the cytoplasmic pool 34. Tavazoie et al. demonstrated that total tRNAGluUUC overexpression enhanced EXOSC2 translation to promote the metastatic progression of breast cancer 5. Our study found a total of 15 tRNA-Ala-AGC isodecoders among the 22 isodecoders of tRNA-Ala-AGC in MDA-MB-468 cells through tRNA high-throughput sequence analysis, which was consistent with existing findings that tRNA expression is preferential and tissue- and cell-specific 35, 36. tRNAs are transported from the nucleus to the cytoplasmic pool, mainly by XPOT, to exert their translational function. Our study further indicated that nucleocytoplasmic transportation of isodecoder tRNA through XPOT was also preferential and selective in TNBC cells. We found that XPOT preferentially affected tRNA-Ala-AGC-10-1 nucleocytoplasmic distribution, but not other tRNA-Ala-AGC isodecoders, through tRNA high-throughput sequencing, which was further confirmed by qRT-PCR. In addition, our tRNA-seq results also revealed that XPOT could preferentially affect the nucleocytoplasmic distribution of tRNA-Glu-TTC-1-1 and tRNA-Glu-TTC-2-1, but not the other isodecoders of tRNA-Glu-TTC. Intriguingly, our study is the first to show that the nucleocytoplasmic distribution of different tRNA isodecoders is distinct in our experimental cells after XPOT knockdown. It has been reported that even C or D loop alterations of the same tRNA can influence the interaction affinity with XPOT in Xenopus oocyte cells 17. tRNAs have the same anticodon with different genome sequences, which are termed isodecoders 37, 38, and the sequence and spatial structure of each tRNA-Ala-AGC isodecoder are distinctive, as shown in Figure S6; therefore, theoretically different tRNA-Ala-AGC isodecoders may have different interaction affinities with XPOT during nucleocytoplasmic transportation, which provides direct evidence to support this hypothesis. Therefore, these studies partially support our novel finding that XPOT could preferentially influence the nucleocytoplasmic distribution of specific tRNA isodecoders. However, the exact underlying mechanism requires further investigation. Interestingly, bioinformatic analysis has reported that one of the tRNA-Ala-AGC isodecoders is related to OS and RFS in breast cancer 3, and tRNA-Glu-TTC was also reported to function as an oncogenic gene in breast cancer. Based on these results, we speculate that XPOT might prefer to preferentially influence the export of oncogenic tRNA to the cytoplasm to execute the corresponding biological functions.

XPOT can transport different tRNAs under different conditions according to cell growth or metabolic requirements 39. In this study, we found that XPOT ensures completion of cytokinesis in TNBC cells, possibly by regulating the translation of the cytokinesis-gene TTC19 by influencing tRNA-Ala-AGC-10-1 nucleocytoplasmic trafficking. Anticodon-AGC can identify the codon GCR (GCT, GCC, GCA) because of codon degeneracy; however, the cognate codon of tRNA-Ala-AGC-10-1 is GCT. tRNA-Ala-AGC-10-1 preferentially identified mRNA enrichment in GCT. Through codon usage preference analysis (calculating all MDA-MB-468 expressed genes whose coding sequences were enriched in GCT) and protein mass spectrometry assays, we identified a series of genes influenced by XPOT and tRNA-Ala-AGC-10-1. Interestingly, given the regulatory role of XPOT in cytokinesis, we found that one cytokinesis-related gene, TTC19, was ranked top-2 among all genes regulated by both XPOT and tRNA-Ala-AGC-10-1. Our experiments demonstrated that XPOT did not influence TTC19 transcription or degradation. The effect of XPOT on TTC19 expression was GCT-dependent. Collectively, these results suggest that XPOT affects TTC19 translation levels depending on tRNA-Ala-AGC-10-1 nuclear transportation to the cytoplasmic pool. Our study reveals a novel mechanism that XPOT affects the translation of specific genes by increasing the nucleocytoplasmic transportation of specific tRNAs to the cytoplasmic pool without influencing tRNA expression. Previous studies support this point, at least partially, showing that cancer-related proteins may have a high frequency of codon usage because of the increased number of homologous tRNAs in the cytoplasmic pool 39. TTC19 is both related to the regulation of cytokinesis and function of mitochondrial complex III according to the annotation in “The Human Gene Database-GeneCards” (https://www.genecards.org/). Previous studies have reported the role of TTC19 in the regulation of respiratory chain complex III 40, 41. However, the exact role and underlying mechanism of TTC19 in cytokinesis remain to be elucidated. Our study is the first to demonstrate that TTC19 is a cytokinesis gene regulated by XPOT through tRNA-Ala-AGC-10-1 and might have an oncogenic function in TNBC.

## Conclusion

In summary, our findings reveal a new XPOT-tRNA-TTC19 pathway that regulates TNBC cytokinesis and development. The XPOT-dependent specific tRNA transportation modulation may provide a novel potential molecular treatment target for TNBC, and it also offers a new perspective on extensive physiological and pathological processes. XPOT, as a regulator of cytokinesis through TTC19, may be a potential novel prognostic and therapeutic biomarker for TNBC.

## Methods

### Human samples

We obtained 12 paired TNBC and adjacent normal tissues from patients who underwent mastectomy or modified radical mastectomy surgery in the Department of Breast Surgery, Renji Hospital, School of Medicine, Shanghai Jiaotong University. All patients signed informed consent before operations. Human samples were collected between 2014 and 2015. All patients enrolled in this study were histopathologically and immunohistochemically diagnosed with TNBC. All procedures were approved by the Ethical Review Committee of the World Health Organization (WHO) Collaborating Center for Research in Human Production (authorised by the Shanghai Municipal Government).

### Animal experiments

The experimental animals were fed and housed in conformance with the guidelines of the Institutional Animal Care and Use Committee of East China Normal University. To evaluate the effect of XPOT and TTC19 on tumour growth, female nude mice were randomly divided into 2 groups and 3 groups (n = 6 per group): shNC group vs. shXPOT group; shNC + vector group vs shXPOT + vector group vs shXPOT + oeTTC19 group. We injected a total of 2 × 10^6^ cells stably expressing the targeted gene combined with 50% Matrigel (356234; Corning, China) + 50% PBS into the left back flank of 5-week-old mice to construct a subcutaneous xenograft model. Then, the tumor maximum and minimum diameter was determined every 5 days using calipers once the tumors were macroscopic in size. The tumor volumes were calculated using the following equation: volume = (length × width^2^) × 0.5. All experimental mice were dissected 30 days after the tumor injection day, and the tumors were acquired and weighed. Finally, the tumors were subjected to formalin fixation, paraffin embedding, and sectioned for IHC analysis.

### Bioinformatic analysis of karyopherins expression in different cancer types

Expression profile of karyopherins in different cancer types comparing with normal tissue was obtained from Oncomine (https://www.oncomine.org/). Besides breast cancer, we also enrolled other cancer types with high incidence like lung cancer, colorectal cancer, gastric cancer, and liver cancer. Several cancer types with low incidence like oligodendroglioma and pleural malignant mesothelioma were also contained.

1. Squamous Cell Lung Carcinoma vs. Normal. Bhattacharjee Lung, Proc Natl Acad Sci U S A, 2001.

2. Colorectal Carcinoma vs. Normal. Hong Colorectal, Clin Exp Metastasis, 2010.

3. Pleural Malignant Mesothelioma vs. Normal. Gordon Mesothelioma, Am J Pathol, 2005.

4. Adrenal Cortex Carcinoma vs. Normal. Giordano Adrenal, Am J Pathol, 2003.

5. Ductal Breast Carcinoma vs. Normal. Richardson Breast 2, Cancer Cell, 2006.

6. Rectal Adenocarcinoma vs. Normal. TCGA Colorectal.

7. Infiltrating Bladder Urothelial Carcinoma vs. Normal. Dyrskjot Bladder 3, Cancer Res, 2004.

8. Granular Renal Cell Carcinoma vs. Normal. Higgins Renal, Am J Pathol, 2003.

9. Gastric Intestinal Type Adenocarcinoma vs. Normal. Chen Gastric, Mol Biol Cell, 2003.

10. Esophageal Squamous Cell Carcinoma vs. Normal. Hu Esophagus, BMC Genomics, 2010.

11. Hepatocellular Carcinoma vs. Normal. Roessler Liver 2, Cancer Res, 2010.

12. Small Cell Lung Carcinoma vs. Normal. Garber Lung, Proc Natl Acad Sci U S A, 2001.

13. Acute Myeloid Leukemia vs. Normal. Andersson Leukemia, Leukemia, 2007.

14. Burkitt's Lymphoma vs. Normal. Basso Lymphoma, Nat Genet, 2005.

15. Oligodendroglioma vs. Normal. Shai Brain, Oncogene, 2003.

### Immunohistochemical staining

TNBC tissue microarrays (TMA) were purchased from Superbiotek (Shanghai, China). In total, 80 paired patient samples were enrolled, including tumor tissue and their counterpart normal tissues. Samples were collected between 2005 and 2012. The average age of this cohort of patients with TNBC was 52 years (range, 18-86 years). The follow-up endpoint was May 2019, and the median survival time was 393.5 months (range, 7 to 180 months). We used TMA to perform immunohistochemical (IHC) staining and IHC score evaluation. The protocol was conducted as previously described 42. XPOT and TTC19 were examined using the corresponding primary antibodies: sc514591 (1:100) (Santa Cruz Biotechnology) and 208751AP (1:50) (Proteintech), respectively. Images were obtained using a fluorescent microscope (Carl Zeiss, Oberkochen, Germany). We evaluated the protein staining score based on the following criteria: a dark brown staining of tumor cells of 81-100% was defined as “++++”; a dark brown staining of tumor cells of 51-80% was defined as “+++”; a dark brown staining of tumor cells of 21-50% was defined as “++”; a dark brown staining of tumor cells of 10-20% was defined as “+”. Two experienced pathologists evaluated the scores, both independently and blindly. We classified tumors staining with “+++” and “++++” into the high expression group, and “+” and “++” into the low expression group, which were utilized for the survival analyses.

### Cell lines culture and regents

All human cell lines, including the normal mammary epithelial cell line HBL100 and TNBC cell lines (MDA-MB-231, MDA-MB-436, MDA-MB-468, BT20, HCC1937, and HCC1806) were purchased from the American Type Culture Collection (ATCC). All cells were cultured in the specified medium according to ATCC protocols. The medium was supplemented with 10% foetal bovine serum (FBS) (Life Technologies, Inc., USA) and 1% penicillin/streptomycin (P/S) (Life Technologies, Inc., USA) in an incubator at 37°C with 5% CO_2_. MDA-MB-468 cells were cultured in a 37°C/non-CO_2_ atmosphere. We tested all cell lines in December 2018 and again every 4 months to verify that mycoplasma was negative by the Shanghai Cancer Institute. Proteasome inhibitor MG132 (133407-82-6; MCE, China) was used as the reagent.

### Stable knockdown cell line construction

We designed and purchased the short harpin RNA targeting the human XPOT sequence and sh-scramble, which were attached to the PLKO-puro lentiviral vector (Gene Pharma, Shanghai, China). Briefly, 4 × 10^5^ MDA-MB-231 and MDA-MB-468 cells were plated in 60-mm dishes. Once the cells were adherent, 1 × 10^6^ of lentivirus was added to the dish after the addition of 5 µg/ml Polybrene (H9268; Sigma-Aldrich, St. Louis, MO). Stable knockdown cells were screened using puromycin (A1113802; Gibco) from 2 µg/ml to 8 µg/ml after transfection for 72 h. The knockdown efficiency was validated by western blotting. The sequence of shXPOT was 5'- GCACAUUCCAUGUGUACUATT-3'.

### Cell transfection

Gene knockdown was performed using Lipofectamine RNAiMAX (Invitrogen, Carlsbad, CA, USA) combined with 150 pmol small interfering RNA (siRNA) constructs or 5 µL of negative control siRNA (Gene Pharma, Shanghai, China). The targeted gene sequences were as follows: siXPOT-1 sense: 5'-GGGACAGUCAUUGAUAGUUTT-3'; antisense: 5'-AACUAUCAAUGACUGUCCCAT-3'; siXPOT-2: sense: 5'-GCACAUUCCAUGUGUACUATT-3'; antisense: 5'-UAGUACACAUGGAAUGUGCTG-3'. siTTC19-2: sense, 5'-GCAGGAGGACAAUGCAAUATT-3'; antisense: 5'-UAUUGCAUUGUCCUCCUGCTT-3'; siTTC19-3, sense: 5'-CUGGCUAUGAAUUCUGCAUTT-3'; antisense: 5'-AUGCAGAAUUCAUAGCCAGTT-3'. TTC19 overexpressing plasmids or their vectors were transfected into XPOT stable knockdown cells using Lipofectamine 2000 (11668-019; Invitrogen). Transfection steps were similar with those used for siRNA transfection.

### Stable overexpression of TTC19 cell line construction

shXPOT and shNC MDA-MB-468 cells were infected with pSLenti-EF1a-EGFP-P2A-CMV-TTC19-3Flag lentivirus (OBiO Technology, Shanghai) and its control vector, respectively. Before virus transfection, XPOT knockdown efficiency was determined by western blotting. TTC19 stably overexpressing cells were acquired by blasticidin selection from 1 to 5 µg/ml when no cells were killed. Western blot was used to observe transfection efficiency. These stable cell lines were for the following animal experiments.

### Cell proliferation detection

5‐Ethynyl‐2‐deoxyuridine assay (EdU) was performed according to the protocol of 5‐ethynyl‐2'‐deoxyuridine (EdU)-labelling/detection pack (Beyotime, Shanghai, China). Fluorescence microscopy was used to observe the cells. Cell CCK-8 and cell colony formation assays were conducted as previously described 43.

### Cell cycle analysis

Cells were transfected with siXPOT or si-TTC19 and scramble RNA for 48 h before incubating with serum-free medium to synchronise cells at the G1/S boundary. After 24 h, the cells were cultured in complete medium for an additional 24 h. The cells were collected and washed twice with cold PBS before incubating with 25 µg/ml RNaseA and 2.5 µg/ml propidium iodide (Dojindo, Kumamoto, Japan) for 30 min in the dark at 4°C, followed by 30 min at 37°C. The stage of the cell cycle was determined using flow cytometry (LSRFortessa; BD Biosciences, USA). Data were analysed using ModFit LT software.

### Cell apoptosis assay

Cells (3 × 10^5^ cells/well) were seeded in a 6-well plate overnight for attachment. siRNAs or plasmids were added to the cells for 48 h, followed by incubating in serum-free medium for another 24 h. To determine the apoptotic cell ratio, the cells were stained using an annexin V-FITC apoptosis detection kit (Dojindo, Kumamoto, Japan) and examined by flow cytometry for analysis. Data were analysed using FlowJo version 10.0.

### RNA isolation and quantitative real time-PCR (qRT-PCR)

Total RNA was extracted according to the manufacturer's instructions using TRIzol reagent (15596018; Thermo Fisher Scientific). RNA (1000 ng) was then converted into cDNA, as previously described. The cycling conditions were as follows: 95°C for 30 s, 40 cycles of 95°C for 5 s, and 60°C for 30 s. The reaction system included 12.5 μL of SYBR Green PCR mix, 1 μL of each primer, and 1 μL of cDNA template in a final volume of 25 μL per reaction (RR820A; Takara, Japan). The 2^-△△Ct^ method was used to analyse the relative gene expression (target gene expression normalised to the expression of the endogenous control gene). The qRT-PCR experiments were performed in triplicate.

### Human tRNA Sequencing

We sequenced tRNA genes in the cytoplasm and nucleus of XPOT stably knocked-down TNBC (MDA-MB-46) cells. Total RNA was isolated from the cytoplasm and nucleus of the targeted cells. Before the sequencing experiment, we checked the integrity and quantity of each RNA sample using agarose gel electrophoresis in a NanoDrop ND-1000 instrument. tRNA was isolated from total RNA using the Denaturing Urea Polyacrylamide Gel Electrophoresis (Urea PAGE) method. Briefly, approximately 2 µg total RNA was resolved on 7.5% urea PAGE gels (7M urea) and recovered within a size window of 60-100 nt for tRNA. The “demethylation” section in rtStarTM tRF&tiRNA Pretreatment Kit (AS-FS-005; Arraystar) was used for tRNA m1A&m3C demethylation treatment. A 50-µL demethylation reaction mixture was prepared according to the manufacturer's protocol and incubated at 37°C for 2 h. Then, 40 µL of nuclease-free water and 10 µL of 5× Stop Buffer were added to terminate the reaction. The demethylated tRNA was purified by phenol-chloroform extraction and ethanol precipitation. Demethylated tRNA was partially hydrolysed according to the Hydro-tRNA seq method, with little modification. tRNA was subjected to limited alkaline hydrolysis in 15 µL of buffer comprised of 10 mM Na_2_CO_3_ and 10 mM NaHCO_3_ (pH 9.7) at 90°C for 7 min. The partially hydrolysed tRNA was dephosphorylated with 10 U calf intestinal alkaline phosphatase (CIP) (M0290L; New England Biolabs) in a 50-µL reaction of 100 mM NaCl, 50 mM Tris-HCl (pH 7.9), 10 mM MgCl_2_, 1 mM DTT, 3 mM Na_2_CO_3_, and 3 mM NaHCO_3_, at 37°C for 1 h. The resulting tRNA was purified with TRIzol reagent and then re-phosphorylated with 10 U T4 polynucleotide kinase (M0201L; New England Biolabs) polynucleotide kinase in a 20-µL reaction mixture containing 70 mM Tris-HCl (pH 7.6), 10 mM MgCl_2_, 5 mM DTT, and 1 mM ATP at 37°C for 1 h. TRIzol reagent purification was performed once more. Partially hydrolysed tRNA fragments were converted to barcoded small RNA sequencing libraries using the NEBNext^®^ Multiplex Small RNA Library Prep Set for Illumina^®^ (E7300L/E7850L; New England Biolabs), according to the manufacturer's instructions. The procedures generally included: (1) 3'-adapter ligation; (2) 3'-adapter blocking; (3) 5'-adapter ligation; (4) reverse transcription; (5) PCR amplification; (6) size selection of ∼140-155 bp PCR amplified fragments (corresponding to ∼19-35nt tRNA fragments) using 6% PolyAcrylamide Gel (PAGE). The completed libraries were qualified and quantified using the Agilent 2100 Bioanalyzer. The DNA fragments in well-mixed libraries were denatured with 0.1 M NaOH to generate single-stranded DNA molecules, and loaded onto the reagent cartridge for 75 cycles (NextSeq 500/550 High-Output v2 kit, FC-404-2005; Illumina) at a loading. Comprehensive data and statistical analyses were obtained using the Arraystar tRNA-seq data analysis package.

To semi-quantitatively analyse tRNA, we performed qRT-PCR based on the results of tRNA-seq. tRNA demethylation was performed as described above. The cycling conditions were as follows: 95°C for 30 s, 40 cycles of 95°C for 5 s, and 60°C for 30 s. tRNA primers were purchased from Arraystar (USA).

### Cytoplasmic and nuclear RNA purification and tRNA qRT-PCR

Cytoplasmic and nuclear RNA were harvested according to the manufacturer's protocol using the Cytoplasmic and Nuclear RNA Purification Kit (Norgen, Canada). Total cellular RNA was isolated as described previously. tRNA demethylation, reverse transcription, and qPCR were performed in conformance with a previous study 44. These procedures required reagents including a demethylation kit, First-Strand cDNA Synthesis Kit, and SYBR (Arraystar, USA). To semi-quantitatively analyse tRNA, we performed qRT-PCR based on the results of tRNA-seq. tRNA demethylation was performed as described above. The cycling conditions were as follows: 95°C for 10 min, 40 cycles of 95°C for 10 s, and 60°C for 60 s. tRNA (tRNA-Ala-AGC-10-1, tRNA-Ala-AGC-1, tRNA-Ala-AGC-3, tRNA-Thr-viviAGT-3-1, and tRNA-Gly-CCC-1-1) primers were purchased from Arraystar (USA).

### Codon usage preference analysis

Based on the NCBI CCD database, the protein-coding nucleic acid sequence (coding sequence) of the genes (all genes detected in our RNA-seq data) was downloaded, and the frequency of codon usage in each protein-corresponding gene was calculated. Then, the frequency was converted into the frequency of each codon in every 1000 nucleic acid bases. We selected genes with the ability to code protein and codon-GCT usage frequency of more than 0.020, while the ratio of GCT score/GCC score and GCT score/GCA score were both >1.

### RNA sequencing

Total RNA was extracted from siNC or siXPOT/MDA-MB-468 cells. The Agilent 2100 Bioanalyzer (Agilent Technologies, Santa Clara, CA, USA) was used to examine RNA integrity. RNA integrity number (RIN) ≥ 7 qualified for subsequent analysis. We used the TruSeq Stranded mRNA LT Sample Prep Kit (Illumina, San Diego, CA, USA) to construct the libraries in accordance with the manufacturer's protocols. Then, we sequenced these libraries on the Illumina sequencing platform (Illumina HiSeq X Ten) and generated 150-bp paired-end reads.

### Protein mass spectrometry

The reagents used in this experiment were as follows: urea, ammonium bicarbonate, dithiothreitol, iodoacetamide, BCA protein quantitative kit, 10 kD ultrafiltration tube, sequencing grade modified trypsin, C18 micro column, formic acid, acetonitrile, and water. First, we extracted the protein supernatant and measured its concentration. Second, we processed the extracted proteins by reduction and alkylation. Third, we enzymatically hydrolysed the processed protein. We also hydrolysed the processed protease into polypeptides and desalinated the polypeptides. Lastly, the samples were tested using Nano Liquid Chromatography-Orbitrap Mass Spectrometry system. The original data were searched and analysed using Proteome Discoverer 2.3 (Thermo Fisher Scientific) software, and the search engine was sequenced. The human protein database was downloaded from UniProt (https://www.uniprot.org/). Its oxidation process is fixed through the modification of cysteine alkylation, the variable modification of methionine oxidation, the deamidation of asparagine/glutamine, and n-terminal acetyl. The precursor mass tolerance was set to 10 ppm, and the fragment mass tolerance was set to 0.02 Da, allowing the Max missed cleavages to 2. FDR (False discovery rate) was set to ≤1%. Data were normalized by shifting the median H/L ratio to 1.

### Construction of synonymous codon mutation plasmids

To obtain synonymous codon mutations in the TTC19 coding sequence (CDS), CDS contains 13 codon GCT. we constructed wild and codon mutation plasmids from OBiO Technology (Shanghai, China): TTC19 wild plasmid, all GCT changing into GCC mutation plasmid, or GCT changing into GCA mutation plasmid. These plasmids were transfected into XPOT stable knockdown or control cells according to the above transfection protocol. After 72 h, western blotting was performed to confirm the XPOT-related codon-dependent translation of TTC19.

### Western blotting

Total protein was extracted and quantified using a BCA protein assay kit (P0010; Beyotime, China). Proteins (20-30 μg) were separated on 10% SDS-polyacrylamide gels and transferred to polyvinylidene difluoride (PVDF) membranes (Millipore, Billerica, MA, USA). The membranes were incubated in 5% non-fat milk diluted with TBST (0.1% Tween-20 in Tris-buffered saline) to block nonspecific binding. The membranes were incubated with primary antibodies at 4°C overnight, and then incubated with horseradish peroxidase-conjugated secondary antibodies (1:2000 dilution in 1% BSA) at room temperature for 1 h. The following antibodies were used: β-catenin (1:5000) (ab32572; Abcam, Cambridge UK), GAPDH (1:1000) (10494-1-AP; Proteintech), tubulin (1:3000) (ab0049; Abways), XPOT (1:100) (sc514591; Santa Cruz Biotechnologies), and TTC19 (1:50) (20875-1-AP; Proteintech).

### Immunofluorescent staining

To determine the role of XPOT or TTC19 in cytokinesis, cells were transfected with siXPOT, siNC, siTTC19, or siNC for 72 h. Then, 4000 cells were seeded into each well of the 12-well chambers (Ibidi, Martinsried, Germany). After adherence, the cells underwent a series of manipulations: fixing with 4% PFA for 15 min, permeabilizing with 1% TritonX-100 for 5 min, and enclosing with 1% BSA for 5 min at room temperature. Enclosed cells were incubated at 4°C overnight with β-tubulin primary antibodies (1:40) (10094-1-AP; Proteintech), and then incubated with secondary antibody conjugated with Alexa Fluor-488 (1:100) (Invitrogen, USA) for 1 h at room temperature. Nuclei were stained with DAPI (Sigma-Aldrich, USA) for 5 min. Finally, we captured the images using confocal microscopy (LSM 510; Carl Zeiss, Oberkochen, Germany).

### Live cell image

Live cell imaging time-lapse video microscopy was used to evaluate cell division. Cells were seeded into 12-well plates and transfected with siRNA targeting XPOT or TTC19. After 72 h, the cells were placed in an incubator (37℃ and >95% humidity) (Nikon, Tokyo, Japan) for the observation of cell division using a Biostation Timelapse system. Differential interference contrast images were captured every 15 min/photo with a 20- objective lens for 10 h continuously. Nikon NIS-Elements software was used to analyse the resulting images.

### RNAscope

Cells were fixed with 10% neutral buffered formalin at room temperature for 30 min, and subsequently treated with 70% ethanol for 10 min, 90% ethanol for 10 min, and 100% ethanol for 10 min. RNAscope *in situ* hybridisation was performed using the RNAscope kit (Advanced Cell Diagnostics, Newark, CA, USA) according to the manufacturer's instructions. We designed a 1ZZ probe named BA-Hs-tRNA-ala-agc-10-1-1zz-st targeting 2-45 customer-provided sequences (Advanced Cell Diagnostics, Newark, CA). The sequence of tRNA-Ala-AGC-10-1 is “GGGGAATTAGCTCAAGTGGTAGAGCGCTTGCTTAGCACGCAAGAGGTAGTGGGATCGATGCCCACATTCTCCACCA”. Fluorescent images were obtained using a confocal microscope (LSM 510; Carl Zeiss, Oberkochen, Germany). MBF Image J was utilised to analyse the fluorescence intensity.

### Generation of TTC19 knockout cells

The TTC19 knockout cell lines were generated using the CRISPR/Cas9 system. CRISPR guide RNA sequences targeting TTC19 were designed by the online software at http://crispr.mit.edu. The sgRNA sequences was cloned into lentiCRISPRv2 (Addgene Plasmid #52961). The sgRNA sequences were as follows:

sgNC 5'-GCGAGGTATTCGGCTCCGCG-3'

sgTTC19 5'-GTTCTGCGCAGCATAGATAC-3'

HEK293T cells were co-transfected with the lentiCRISPRv2 and packaging plasmids pMD2.G and psPAX2. About 48h later, lentivirus was collected. MDA-MB-231 cells were infected using the lentivirus for 12h and blasticidin-resistant single cells were plated in a 96-well dish to screen for positive monoclonal cells.

### Bioinformatic analysis

Survival analysis of breast cancer patients was referenced from The Cancer Genome Atlas (TCGA) and Kaplan-Meier plotter (http://kmplot.com/analysis). Patients in TCGA TNBC cohort were separated into high and low XPOT/TTC19 expression group with cutoff selected by R package "maxstat". We chose the best cutoff for Kaplan-Meier analysis of breast cancer patients from Kaplan-Meier plotter. TCGA and GEO databases (GSE13650) was used to compare XPOT expression levels between triple negative breast cancer tissue and normal tissue.

### Graphic and statistical analyses

All the data and graphic images were analysed using GraphPad Prism version 8.0 (San Diego, USA). Categorical variables were analysed using Pearson's Chi-square test or Fisher's exact test. Two-sided Student's *t*-test or one-way analysis of variance (ANOVA) was used to analyse the numerical data comparison. Kaplan-Meier plot and log rank test were used to observe the correlation between TTC19 and XPOT expression and survival. Multivariable cox analysis was used for evaluating relationship between prognosis and potential factors. Error bars denote the mean ± standard deviation (SD). Heatmaps were plotted using R (version 3.6.1). Adobe Illustrator (version 21.0.0.0) was used to organise all the images. Statistical significance was defined as two-side *p* < 0.05.

## Supplementary Material

Supplementary figures and table.

Supplementary appendix 1: tRNA-Ala-AGC isodecoders.

Supplementary appendix 2.

## Figures and Tables

**Table 1:** Differential distribution of nucleus and cytoplasmic tRNA isodecoders after XPOT depletion in TNBC cells

tRNA isodecoder	Ratio= 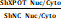
tRNA-Thr-AGT-3-1	2.042221128
tRNA-Ala-AGC-10-1	1.999167172
tRNA-Glu-TTC-1-1	1.658461909
tRNA-Arg-CCT-1-1	1.524281421
tRNA-Glu-TTC-2-1	1.480488632
tRNA-Cys-GCA-5-1	1.477380601
tRNA-Ile-AAT-3-1	1.455185042
tRNA-Val-CAC-5-1	1.378688966
tRNA-Ser-AGA-5-1	1.322156666
tRNA-Cys-GCA-1-1	1.304067958
tRNA-Leu-TAG-3-1	1.258855669
tRNA-Leu-TAA-2-1	1.209773187
tRNA-Lys-CTT-2-1	1.19750431
tRNA-Gly-CCC-1-1	1.145757275

Nuc: nucleus, Cyto: cytoplasm
