# Supplementary material for: XPOT Disruption Suppresses TNBC Growth through Inhibition of Specific tRNA Nuclear Exportation and TTC19 Expression to Induce Cytokinesis Failure
Source: Int J Biol Sci. 2023 Oct 24;19(16):5319–36. doi: 10.7150/ijbs.85006 (PMC10620816; doi:10.7150/ijbs.85006)
Supplement: Supplementary file 1 — Supplementary figures and table. [file ijbsv19p5319s1.pdf]

Supplementary figures and figure legends

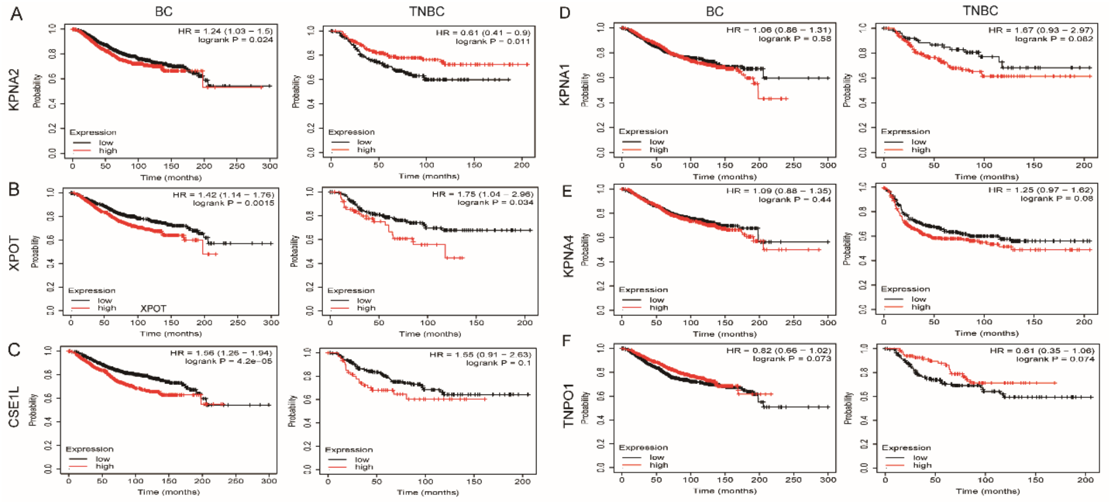

Supplementary Fig 1. Kaplan-Meier overall survival Analyses of top rank ordered karyopherins in breast cancer dataset (Oncomine Richardson Breast 2) in BC (left) and TNBC (right) from the website Kaplan Meier Plotter. A. KPNA2 B. XPOT C. CSE1L D. KPNA1 E. KPNA4 F. TNPO1. BC, breast cancer; TNBC, triple negative breast cancer.

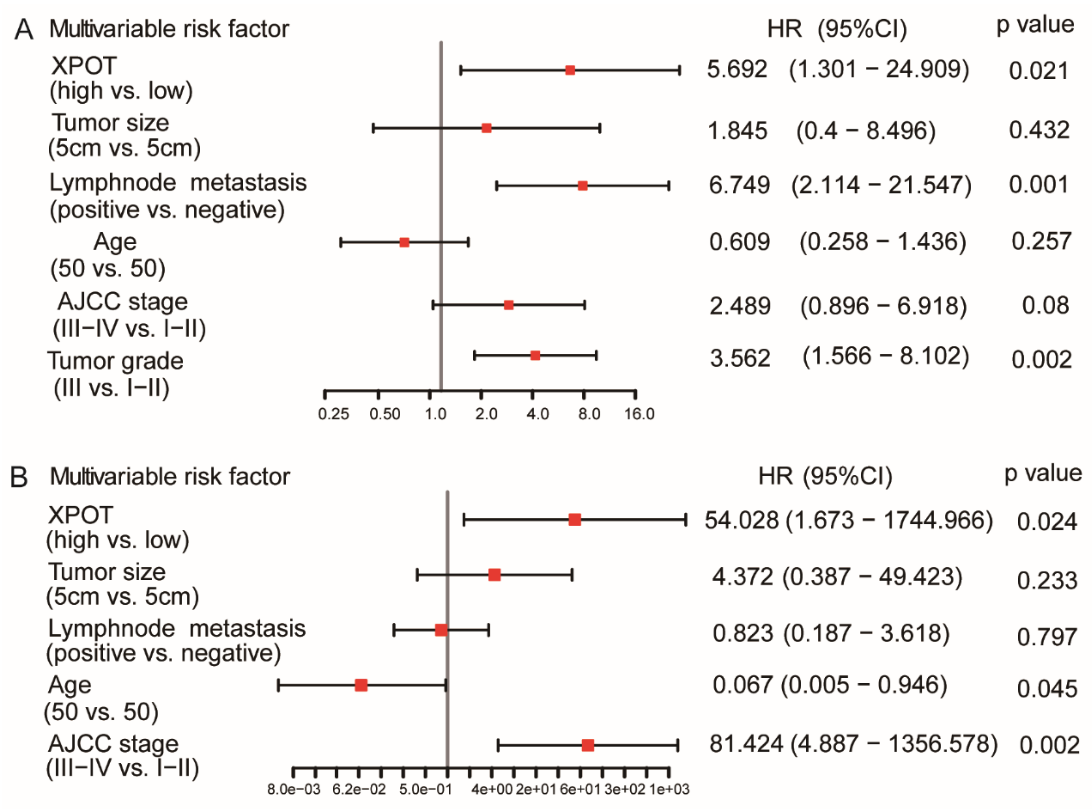

Supplementary Fig 2. Multivariate Cox regression analysis. A and B. Multivariate Cox regression analysis of clinicopathologic factors for TNBC overall survival based on TNBC tissue microarray (A) and TCGA (B), CI, confidence interval; HR, Hazard Ratio.

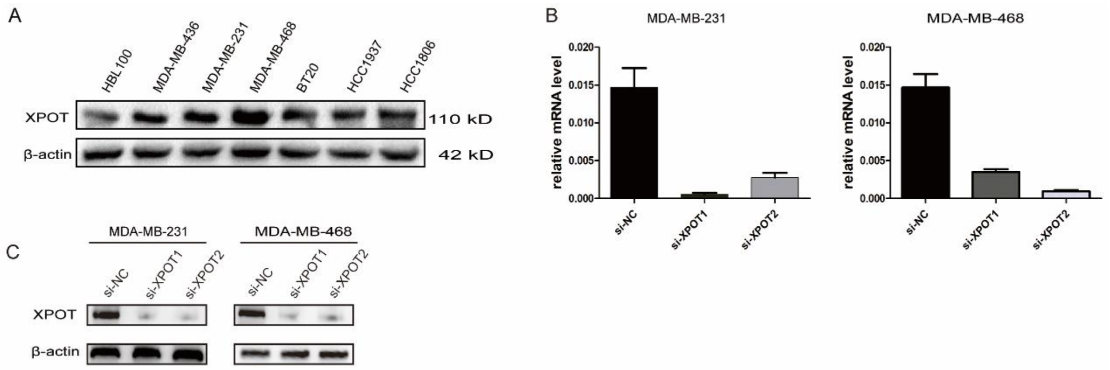

Supplementary Fig 3. Expression of XPOT. A. Western blot analysis of XPOT expression level in 6 TNBC cell lines and normal mammary epithelial cell line HBL100. B. Relative mRNA level of XPOT knockdown efficiency mediated by small interference RNA in MDA-MB-231 and MDA-MB-468 cells. C. XPOT knockdown efficiency was testified by western blot analysis mediated by siRNA in MDA-MB-231 and MDA-MB-468 cells.

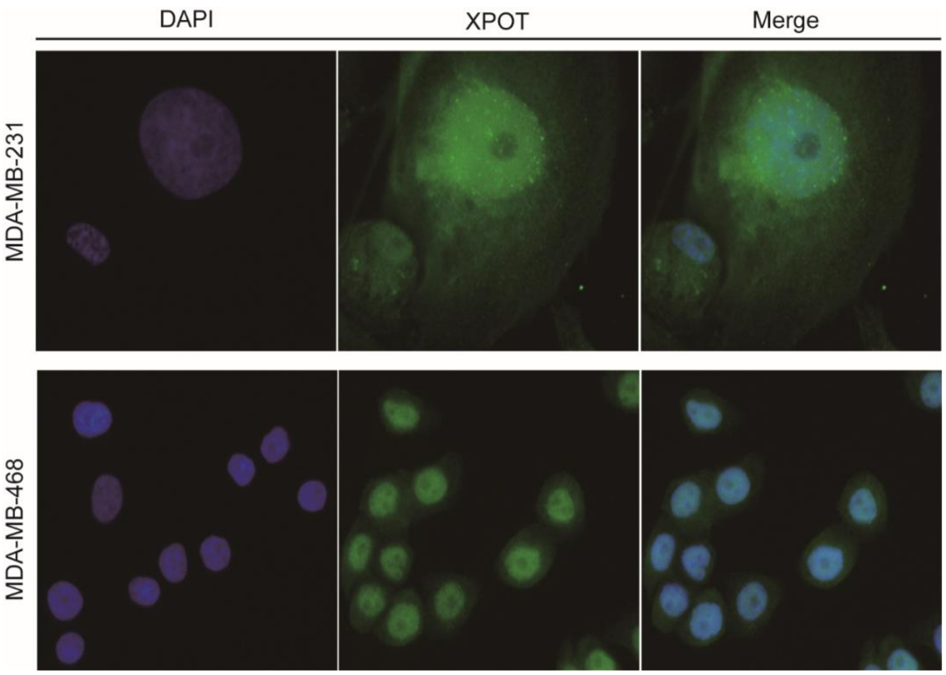

Supplementary Fig 4. XPOT localization in TNBC cells. Representative image of XPOT expression in MDA-MB-231 by immunofluorescence staining.

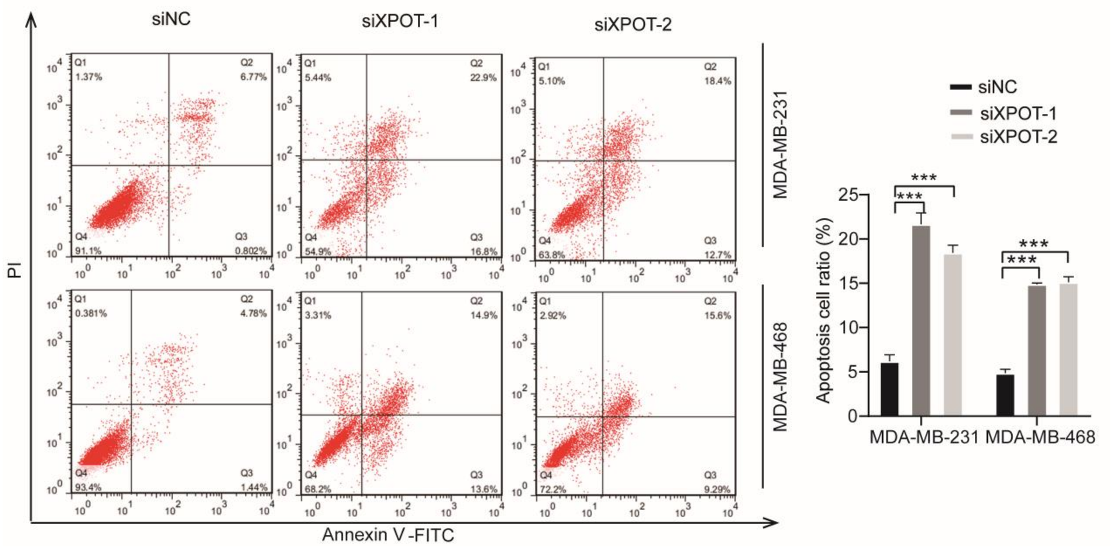

Supplementary Fig 5. XPOT resists apoptosis of TNBC cells. Representative images and quantitative analysis of cells apoptosis assay in MDA-MB-468 and MDA-MB-231 cells. Values were mean ± SD (n=3). (Student's t-test). ***P< 0.001.

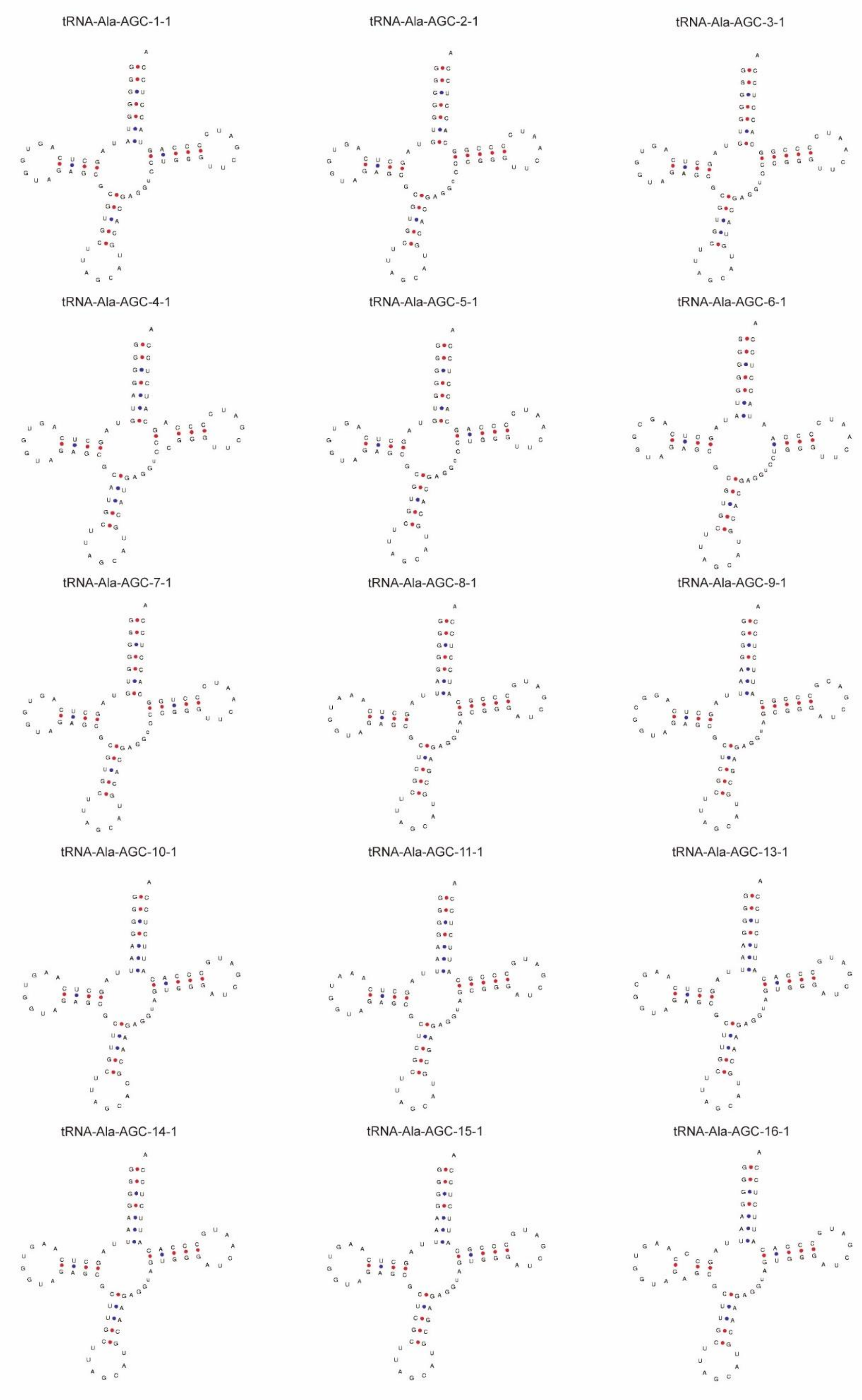

Supplementary Fig 6. Structure of tRNA-Ala-AGC-10-1 isodecoders in MAD-MB-468 cells.

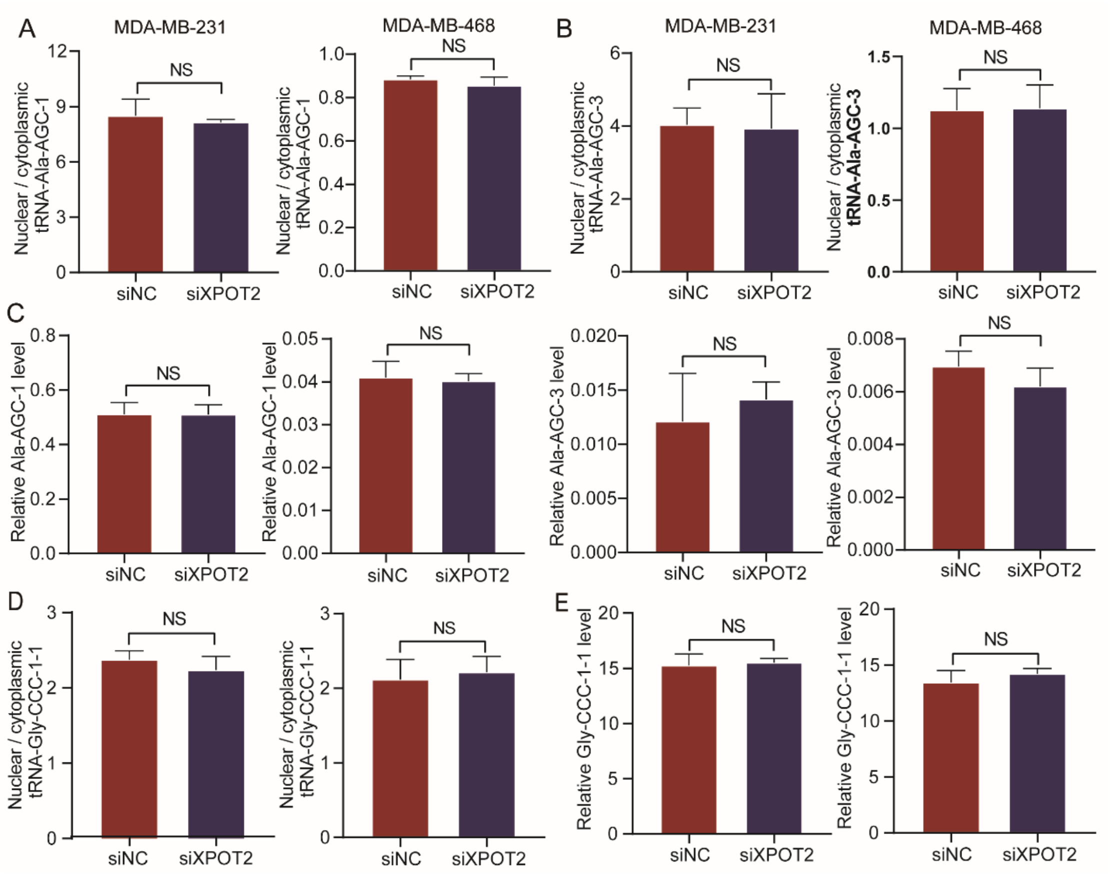

Supplementary Fig 7. XPOT mediates other tRNAs transporting fromg nucleus to cytoplasm. A. Nuclear/cytoplasmic tRNA ratio of tRNA-Ala-AGC-1 between siNC and siXPOT/MDA-MB-231 or MDA-MB-468 cells using RT-PCR assay. Values were mean ± SD (n=3). (Student's t-test). B. Nuclear/cytoplasmic tRNA ratio of tRNA- Ala-AGC-3 between siNC and siXPOT/MDA-MB-231 or MDA-MB-468 cells using RT-PCR assay. Values were mean ± SD (n=3). (Student's t-test). C. RT-PCR assay for total relative tRNA- Ala-AGC-1 and tRNA- Ala-AGC-3 aboundance in MDA-MB-231 and MDA-MB-468 cells. Values were mean ± SD (n=3). (Student's t-test). D. RT-PCR assay for total relative tRNA-Gly-CCC-1-1 aboundance in MDA-MB-231 and MDA-MB-468 cells. Values were mean ± SD (n=3). (Student's t-test). E. RT-PCR assay for total relative tRNA-Gly-CCC-1-1 aboundance in MDA-MB-231 and MDA-MB-468 cells. Values were mean ± SD (n=3). (Student's t-test). NS, no significance.

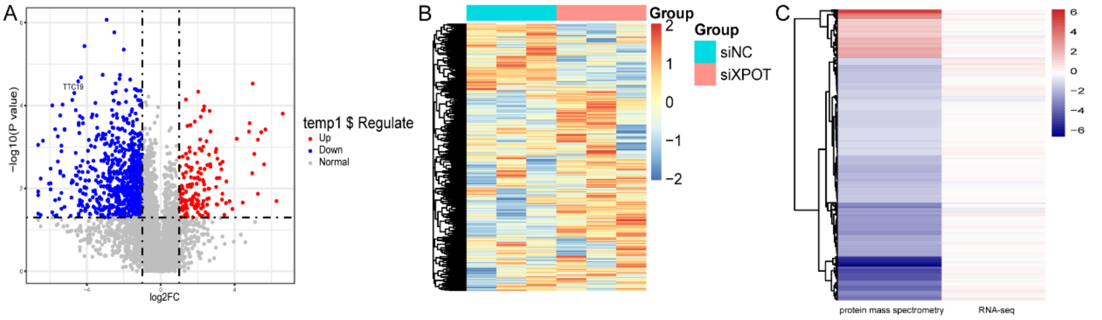

Supplementary Fig 8. Identifying of genes with decreased protein expression while unchanged in mRNA expression after XPOT knockdown. A. Volcano plot showing fold changes (x-axis) and corresponding p values (y-axis) of protein expression between siXPOT and control siNC MDA-MB-468 cells. Compared to siNC group cells, totally 270 proteins were upregulated and 736 proteins were downregulated in siXPOT group cells. Upregulated proteins are defined as Fold change >1.25, p<0.05; Downregulated proteins are defined as Fold change <-1.25, p<0.05. B. Heatmap of mRNAs expression of genes in siNC and siXPOT/MDA-MB-468 cells identified by RNA-Seq. We dectected a totall of 14330 expressed genes in MDA-MB-468 cells. C. Heatmap of genes with downregulated proteins expression while with unchanged mRNA expression in siXPOT group compared with control cells. We totally found 585 eligeble genes. Fold change <-1.25, p<0.05.

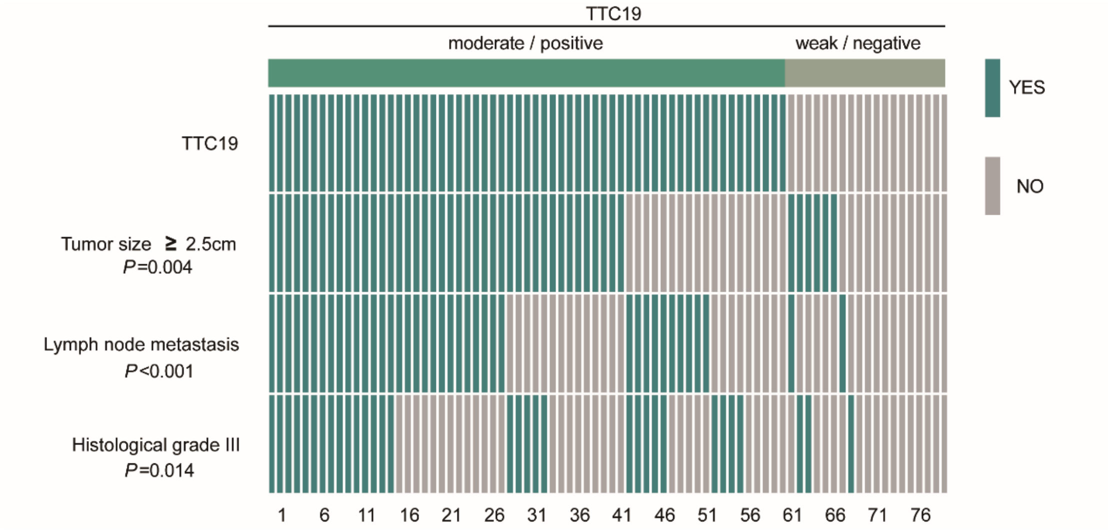

Supplementary Fig 9. Clinical pathological features of TTC19. Correlation analysis of TTC19 expression with tumour size, lymph node metastasis and (TNM) stages of patients with TNBC from tissue microarray (Fisher's exact test).

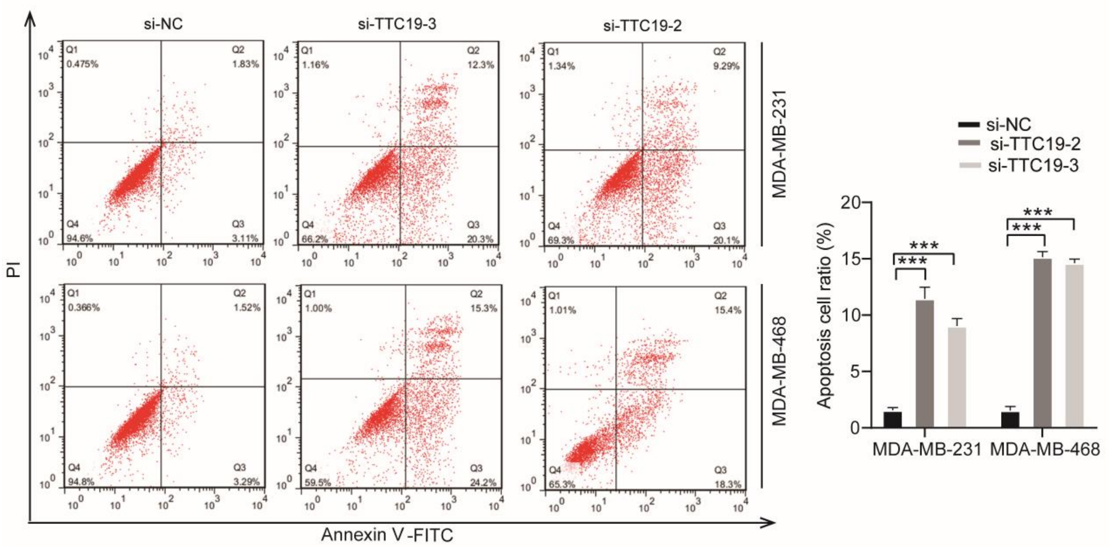

Supplementary Fig 10. TTC19 resisted TNBC apoptosis. Representative images and quantitative of cell apoptosis assay for the effect of TTC19 knockdown in MDA-MB-231 and MDA-MB-468 cells. Values were mean ± SD (n=3). (Student's t-test). ***P< 0.001.

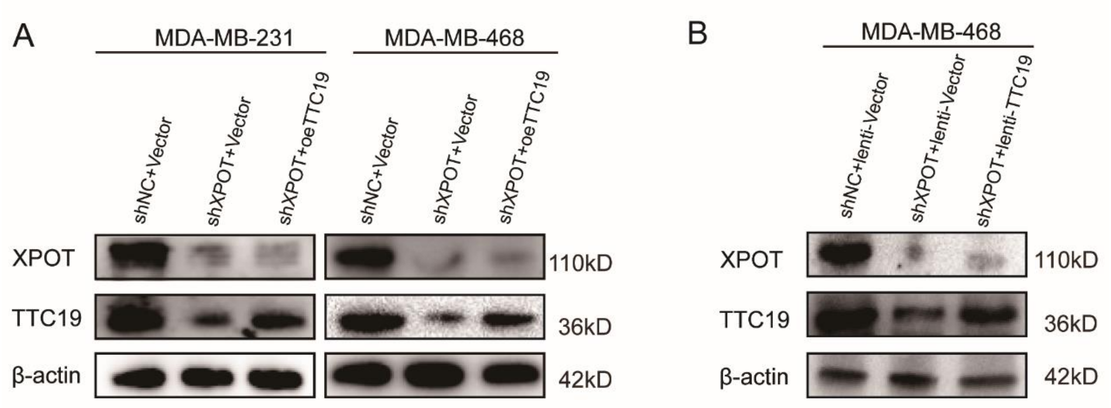

Supplementary Fig 11. Expression of TTC19 in shXPOT TNBC cells. A. Western blot analysis of TTC19 expression in control and XPOT - knockdown (MDA-MB-231 and MDA-MB-468) cells transfected with TTC19 overexpression or control vector. B. Western blot analysis of TTC19 expression in shXPOT/MDA-MB-468 cells transfected with lentivirus-TTC19 or lentivirus-vector.

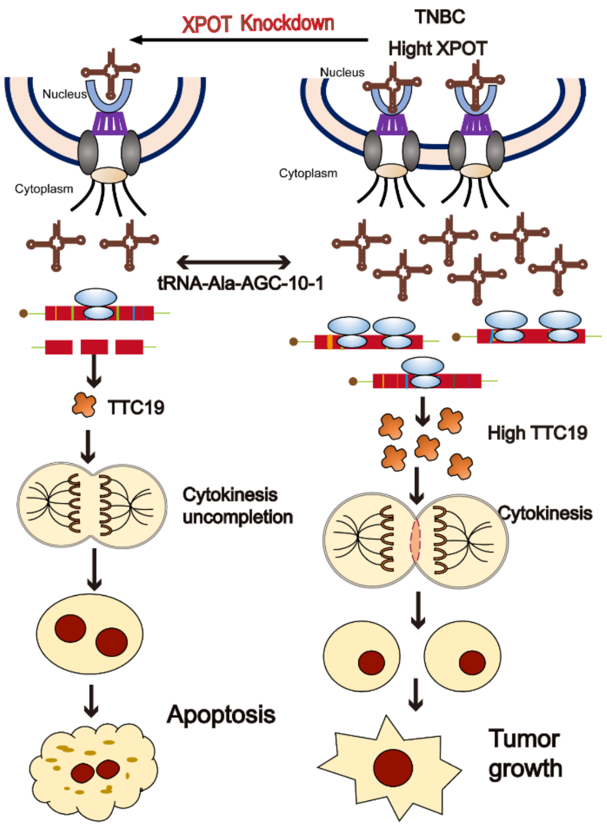

Suppelmentary Fig 12. The mechanistic diagram of XPOT enhancing nuclear export tRNA-Ala-AGC-10-1 to orchestrate faithful cytokinesis in TNBC growth.

1. Supplementary tables

Supplementary Table 1 Primers used for RT- PCR in this study

Primer name	Primer sequence (5'-3')
18S-F	TGCGAGTACTCAACACCAACA
18S-R	GCATATCTTCGGCCCACA
XPOT-F	CCCAACCAGAGAAGACCTTTAT
XPOT-R	ACCAACTCTGAATCAATAGCCA
U6-F	GCTCGCTTCGGCAGCACATATAC
U6-R	CGAATTTGCGTGTCATCCTTGCG
